# Structural basis for broad neutralization of HIV-1 through the molecular recognition of 10E8 helical epitope at the membrane interface

**DOI:** 10.1038/srep38177

**Published:** 2016-12-01

**Authors:** Edurne Rujas, Jose M. M. Caaveiro, Angélica Partida-Hanon, Naveed Gulzar, Koldo Morante, Beatriz Apellániz, Miguel García-Porras, Marta Bruix, Kouhei Tsumoto, Jamie K. Scott, M. Ángeles Jiménez, José L. Nieva

**Affiliations:** 1 Biophysics Unit (CSIC, UPV/EHU) and Department of Biochemistry and Molecular Biology, University of the Basque Country, P.O. Box 644, 48080 Bilbao, Spain; 2 Department of Bioengineering, Graduate School of Engineering, The University of Tokyo, Bunkyo-ku, Tokyo, Japan; 3 Institute of Medical Science, The University of Tokyo, Tokyo, Japan; 4 Institute of Physical Chemistry “Rocasolano” (IQFR-CSIC), Serrano 119, E-28006 Madrid, Spain; 5 Department of Molecular Biology and Biochemistry, Simon Fraser University, Burnaby, Canada; 6 Faculty of Health Sciences, Simon Fraser University, Burnaby, Canada

## Abstract

The mechanism by which the HIV-1 MPER epitope is recognized by the potent neutralizing antibody 10E8 at membrane interfaces remains poorly understood. To solve this problem, we have optimized a 10E8 peptide epitope and analyzed the structure and binding activities of the antibody in membrane and membrane-like environments. The X-ray crystal structure of the Fab-peptide complex in detergents revealed for the first time that the epitope of 10E8 comprises a continuous helix spanning the gp41 MPER/transmembrane domain junction (MPER-N-TMD; Env residues 671–687). The MPER-N-TMD helix projects beyond the tip of the heavy-chain complementarity determining region 3 loop, indicating that the antibody sits parallel to the plane of the membrane in binding the native epitope. Biophysical, biochemical and mutational analyses demonstrated that strengthening the affinity of 10E8 for the TMD helix in a membrane environment, correlated with its neutralizing potency. Our research clarifies the molecular mechanisms underlying broad neutralization of HIV-1 by 10E8, and the structure of its natural epitope. The conclusions of our research will guide future vaccine-design strategies targeting MPER.

The 10E8 antibody achieves potent and broad HIV-1 neutralization by targeting the membrane-proximal external region of gp41 (MPER)^1^,^2^. It appears that this potency is developed after extensive somatic hypermutation of the heavy-chain complementarity determining regions 2 and 3 (CDRH2 and CDRH3, respectively)^3^. The exceptionally high degree of conservation of the MPER sequence^4^,^5^ justifies immunotherapeutic approaches based on the 10E8 antibody^6^,^7^,^8^,^9^,^10^. Supporting its functional activity *in vivo*, 10E8 confers complete protection to rhesus macaques against infection by a simian immunodeficiency virus-HIV chimera^6^. Remarkably, the 10E8 antibody is less effective than many anti-gp120 antibodies in neutralization assays, but provides the strongest protection *in vivo*^7^. Also, consistent with a higher degree of cross-reactivity, 10E8 neutralizes SIVs that infect chimpanzees and gorillas with nanomolar potency^8^, surpassing the efficacy of other broadly neutralizing antibodies directed against different vulnerability regions on Env. Engineered versions of the 10E8 with improved solubility have been recently reported^10^, a finding that broadens the therapeutic potential of this broadly neutralizing antibody^6^,^9^.

In addition, the advantageous properties of 10E8 could be employed for the development of anti-HIV-1 vaccines^4^,^5^,^11^,^12^. However, an important hurdle in this research effort is the observation that the functional properties of 10E8 are not adequately explained by its binding properties to MPER epitope peptides^1^,^2^. In comparison to other MPER-specific neutralizing antibodies, 10E8 exhibits greater potency (mean IC_50_ value below 1 μg/mL) and lacks the lipid binding and auto-reactivity properties previously thought to limit the usefulness of MPER in the conception of an effective HIV-1 vaccine^1^. However, 10E8 binding affinities have been reported to be lower than those of the less-potent neutralizing antibodies, 2F5 and 4E10^1^. Thus, the comparatively poor performance of 10E8 remains at odds with the expectation that tight binding between antibody and epitope underpins the potent biological activity of this antibody, either by stabilizing the MPER domain in a conformation that is incompatible with membrane fusion, or by ensuring the full occupancy required to completely inactivate Env trimers^2^.

Moreover, some mutations in 10E8’s CDRH3 affect viral neutralization more strongly than binding to MPER (*e.g*. Trp100b_HC_Ala), whereas other mutations reduce binding significantly without markedly affecting the neutralizing activity of the antibody (*e.g.* Pro100f_HC_Ala)^1^. Inspection of the crystal structure of the 10E8 Fab in complex with an MPER peptide further reveals this incongruity^1^. The quantification of the interaction surface at the apex of the CDRH3 loop (Trp100b_HC_) performed with PISA indicated that only a small fraction of the surface of this residue (<15%) directly contacts the peptide, whereas the majority of it remains exposed to the solvent. This observation could explain why substitutions of this residue do not abrogate engagement to MPER peptide; however they do not explain its critical role in neutralization. These puzzling observations have obscured the underlying 10E8 mechanism of action, and have thwarted a faithful definition of the antigen structure mediating the biological activity of this important antibody.

In this study, we have elucidated the structure of the 10E8 Fab in complex with a peptide antigen whose affinity has been optimized by the addition of native residues belonging to the gp41 transmembrane domain (TMD)^13^. This peptide has been termed 10E8ep. The dissection of the structural and energetic factors governing the recognition of the epitope in membrane environments explains the potent neutralization capabilities of the antibody. The full-length Env trimer in complex with 10E8 has been recently solved by cryogenic electron microscopy (cryo-EM)^14^. However, the limited resolution (8.8 Å) prevented getting an atomistic understanding of the interactions. Our crystallographic data enhances our understanding of recognition of the 10E8 epitope in the membrane in light of the cryo-EM structure^14^, and past literature^1^,^10^,^15^,^16^. We propose that the helical scaffold of the MPER/TMD region of gp41, strengthened by nonpolar interactions with membrane lipids, is of biological relevance for the generation of potent anti-MPER broadly neutralizing antibodies.

## Results

### Design and characterization of an optimized peptide epitope for 10E8

**Figure 1:**
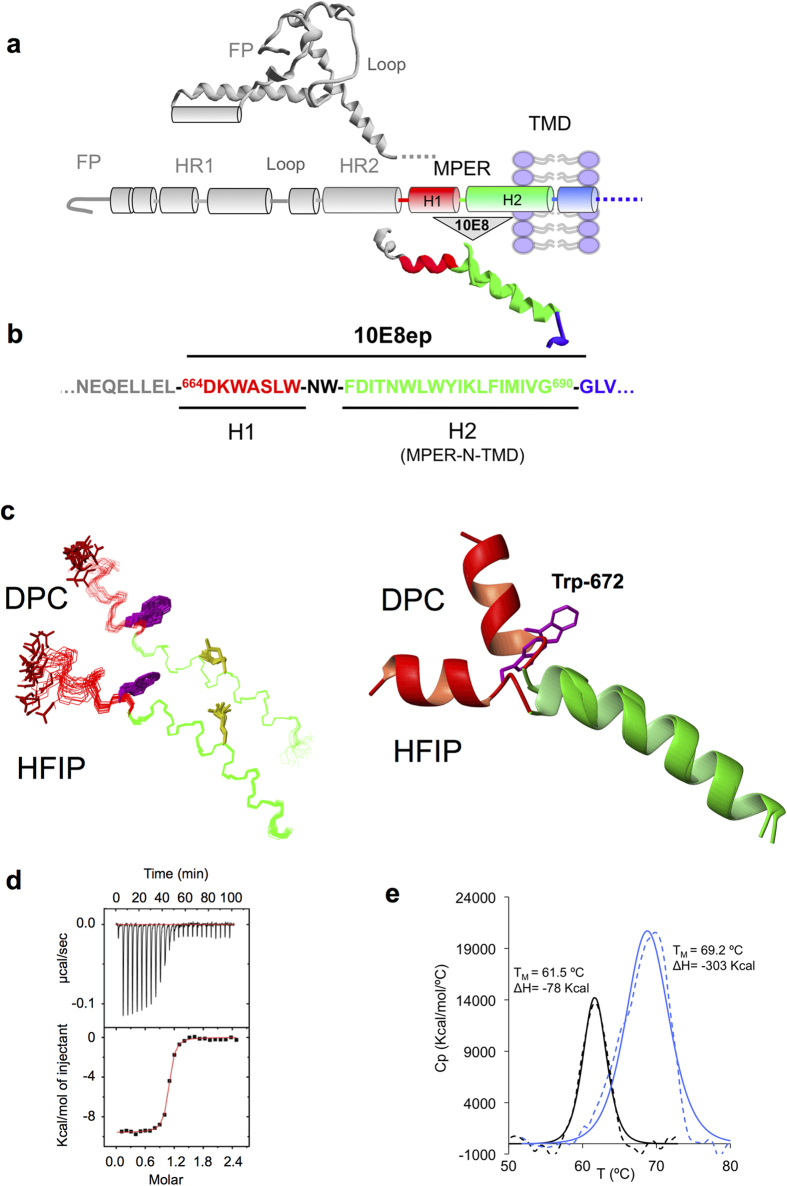
Structure of 10E8ep peptide and energetics of binding to Fab 10E8 in membrane mimetics. **(a)** Structural elements of gp41. The ectodomain pre-fusion structure of gp41 (PDB accession code: 4TVP) is depicted in a ribbon and tube representation above the diagram illustrating the relative positions of the most important constituents in the sequence: FP, fusion peptide; HR1 and HR2, amino- and carboxy-terminal heptad-repeat regions, respectively (helical ribbons), Loop, the region between HR1 and HR2 bearing a short, disulfide-bridged loop; MPER, membrane-proximal external region; TMD transmembrane domain. The ribbon structure below the diagram was modeled by superposing the NMR structures of the MPERp and CpreTM peptides solved in DPC micelles (PDB accession codes 2M8O and 2MG3, respectively), and supports the existence of two distinct helical segments: H1 and H2. **(b)** Env sequence covered by the 10E8ep peptide. **(c)** Structures adopted by 10E8ep in 25% (v/v) HFIP or 20 mM DPC. Left: The 20 lowest target function conformers overlaid onto the backbone atoms of residues 675-691. Lateral side-chain of Asp664, Trp672 and Lys683 residues are depicted in red, green and yellow, respectively. Right: Superposition of representative NMR structures for 10E8p in HFIP and in DPC displayed in ribbon representation. **(d)** Binding isotherm of the 10E8ep peptide to Fab 10E8 as measured by ITC. The upper panel indicates the heat released upon consecutive injections of 10 μL of peptide solution (40 μM) into Fab solution (3 μM) in the calorimeter cell; the lower panel depicts the integrated heats (symbols) and non-linear least-squares fit (line) of the data to a one-site binding model using ORIGIN 7.0. The thermodynamic parameters of binding are listed in Table 1. (**e**) Thermal stability of the Fab-peptide complex by DSC. DSC thermograms (dotted lines) for 10E8 Fab before (black) and after titration with peptide (blue) are shown. Unfolding of the Fab shows a single thermal transition in both cases. The midpoints of the thermal unfolding (*T*_M_) and the associated enthalpies (Δ*H*) are given. The values of *T*_M_ and Δ*H* were obtained by curve fitting using ORIGIN 7.0 (solid lines).

The existence of the continuous H2 α-helix at the MPER/TMD junction (Fig. 1a), termed “MPER-N-TMD” in our recent work^13^, revised models which assumed that the interfacial MPER helix bends at position Lys683 to promote the insertion of the TMD perpendicular to the plane of the membrane^17^,^18^,^19^,^20^,^21^,^22^. We hypothesized that peptides derived from the region MPER-N-TMD might function as a helical scaffold to increase the affinity of antibodies targeting the C-terminal subregion of the MPER^13^. We therefore designed the peptide 10E8ep, *KKKK*-^664^D**K**WA**S**LW-**NW-F**DI**T**NW**LW**YI**K**LFIMIVG^690^-*KKKKK*, to cover the entire length of the H1-H2 helices (Fig. 1b), and to include all the residues reported to establish contacts with the antibody (in bold^1^).

To confirm that the peptide structure contains two α-helical segments, we first analyzed 10E8ep in a membrane-mimetic environment using solution NMR (Fig. 1c). The ^1^H and ^13^C NMR signals of 10E8ep were assigned in the presence of dodecylphophocholine (DPC) micelles of or in the presence of 25% 1,1,1,3,3,3-hexafluoro-2-propanol (HFIP) at pH 7.0 and 25 °C (see Supplementary Methods). Preliminary analyses using native-like MPER sequences indicated that addition of the solubilization Lys-tags was required to obtain reliable spectroscopic information. Moreover, 10E8ep spectra recorded in a mixture of non-deuterated DPC / deuterated DPC-d38 demonstrated that the peptide interacts with the DPC micelle and that the aromatic side chains are immersed into the micelle (not shown). Thus, the 10E8ep/DPC system seems to comprise a *bona fide* surrogate for the antigenic structure recognized by the antibody at membrane interfaces.

The sign and magnitude of the deviations from random-coil values displayed by chemical shifts for most H_α_ protons and C_α_ carbons, and the set of observed non-sequential NOEs provided conclusive evidence for the adoption of helical structures in both media (Supplementary Figs 1 & 2). Structure calculations were further performed to visualize the features of the structure adopted by 10E8ep in the membrane mimics (Fig. 1c). Excluding the Lys solubility tags and Asp664, the resulting structure ensembles were well defined, as indicated by the small RSMD values for the backbone atoms of residues 665–690 (0.7 ± 0.2 Å in HFIP, and 0.7 ± 0.2 Å in DPC; see Supplementary Table 1). Confirming the structural motif inferred from the previous reconstitution process^13^, these helical structures exhibited a kink at position ^671^NW^672^. The variability on the orientation of the two helices within each structural ensemble and the prominent changes in the direction of the helix axis at this position in two selected structures can be appreciated in Fig. 1c (left and right panels, respectively). However, we note that definition of the dihedral angles in a dynamically variable hinge region must be assumed with caution.

To determine the binding signature and the stability of complexes between Fab and the 10E8ep peptide in a membrane-mimetic environment, high-resolution thermodynamic techniques in the presence of DPC micelles were employed (Fig. 1d,e). Titration of 10E8 Fab with the 10E8ep peptide produced a high-affinity value of 9.6 ± 1.0 nM (Fig. 1d and Table 1). The binding free energy (Δ*G°* = −10.9 ± 0.1 kcal mol^−1^) resulted from favorable non-covalent interactions between epitope and antibody (Δ*H°* = −9.6 ± 0.1 kcal mol^−1^), and also from the entropic component which contributed little but positively to the affinity (−*T*Δ*S°* = −1.3 ± 0.2 kcal mol^−1^). The thermal stability of the Fab in the presence and absence of 10E8ep was examined by differential scanning calorimetry (DSC) (Fig. 1e). The data clearly demonstrated that the antibody is stabilized in the complex with peptide with respect to the unbound form. The melting temperature (*T*_*M*_, representing the unfolding mid-point) for the complex increases remarkably with respect to that for the unbound form of the Fab (Δ*T*_*M*_ = 7.7 ± 1.0 °C). In addition, the unfolding enthalpy (Δ*H°*_*DSC*_) of the Fab was significantly increased upon formation of the complex with the peptide (ΔΔ*H°* = 225 kcal mol^−1^). Thus, the thermodynamic analysis indicates that the 10E8ep peptide binds with high-affinity and specificity to the Fab in the presence of DPC micelles.

### Crystal structure of the 10E8ep peptide-10E8 Fab complex

**Figure 2:**
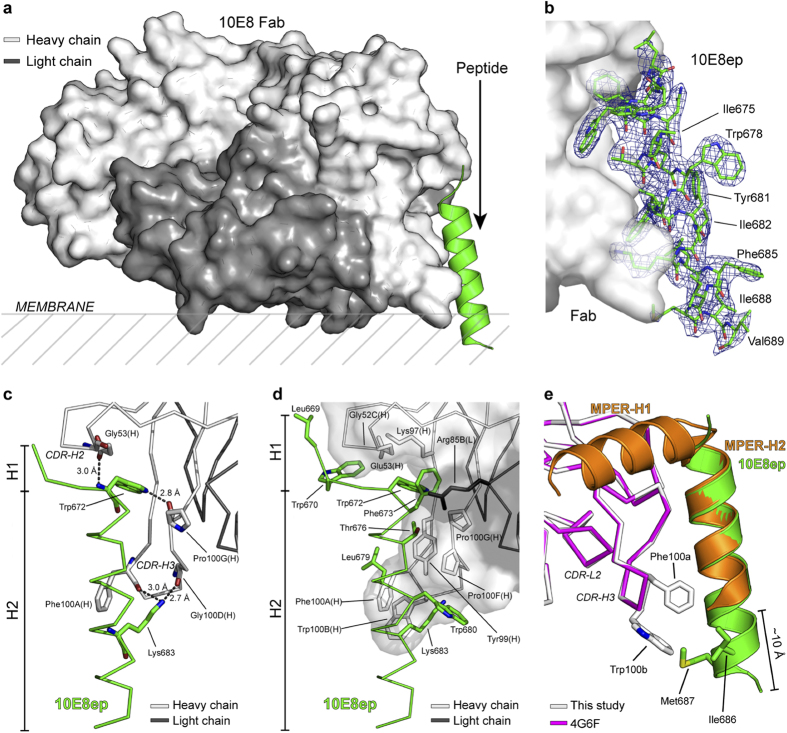
Crystal structure of the complex between Fab 10E8 and the 10E8ep peptide determined at 2.4 Å resolution in the presence of DPC. (**a**) Overall structure of the complex. The surface of the heavy and light chains of the Fab are shown in light and dark gray, respectively. The peptide 10E8ep is depicted as a green helix. The hypothetical location of the viral membrane is shown (see Fig. 4 for more details). (**b**) Sigma-A weighted electron density map (blue mesh) of the peptide 10E8ep bound to the Fab at a contouring level of σ = 1.0. The peptide is depicted as green sticks. Numerous peptide hydrophobic residues (labeled) remain exposed even after engagement with the Fab. (**c**) H-bond network between peptide and Fab. Bond distances are shown. The peptide and Fab are depicted in green and gray sticks, respectively. (**d**) Interaction surface between peptide and Fab. The majority of interacting residues are non-polar. (**e**) Comparison of the conformation of the MPER peptide (PDB entry code 4G6F) and the optimized peptide (this study) bound to 10E8. The Fab and peptide from the previously published structure are depicted in magenta and orange, respectively. The Fab and peptide from this study are shown in light gray and green, respectively. Side-chains of residues Phe100a_HC_ and Trp100b_HC_ of the Fab, and Ile686 and Met687 of the peptide are depicted to illustrate additional interactions.

The X-ray crystal structure of the complex between peptide and Fab in the presence of DPC micelles was determined at 2.4 Å resolution (Fig. 2a, Supplementary Table 2). The model comprised residues 1–215 (heavy chain) and 2–210 (light-chain) of the Fab, and residues 669–689 of 10E8ep. Residues 664–668 of the peptide were not observed in the electron density because of dynamic disorder, consistent with the idea that the H1 helix is not essential for the recognition of Env by this antibody (Fig. 2b–e, Supplementary Fig. S3)^1^,^14^. The region H2 adopted a helical conformation, which is very well conserved among all the structures available for 10E8 and the unbound form (Supplementary Fig. S3a,b)^1^,^10^. The last residue of the H2 region of 10E8ep, Gly690, and the additional Lys residues flanking the sequence of the peptide were not observed in the electron density, reflecting dynamic disorder. The interaction area between Fab and 10E8ep covers only 31% of the surface of the peptide as calculated with the PISA server, leaving numerous aromatic and hydrophobic residues prone to interact with other peptide moieties in the crystal (Supplementary Fig. 4). The assembly of peptides in a “micelle-like” aggregate at crystal-contact regions minimizes the degree of exposure of these hydrophobic residues. In addition, the antibody exhibited a large degree of flexibility at the elbow region between the variable and constant regions with respect to the previous crystal structures with a canonical MPER epitope bound^1^,^10^, or the unbound form^3^ (Supplementary Fig. 3)^23^. We suggest that the flexibility of the elbow region can facilitate the accessibility of the native gp41 epitope, being partially restricted by the gp120 component of Env and by the viral membrane^1^,^14^.

The bound peptide adopts a canonical α-helix throughout the sequence, except for residues at the N-terminal region ^669^LWN^671^ (Fig. 2c and d). In contrast to the kink observed in the unbound peptide, these residues adopt an extended conformation in contact with the antibody. This is consistent with the conformational variability observed in this area^13^,^20^,^24^,^25^. Importantly, no evidence of a kink at position Lys683 was found, in agreement with the solution structure of the peptide determined by NMR (Supplementary Fig. 3) and previous results^13^. The 10E8 Fab is perfectly adapted to recognize the α-helical structure of the peptide as evidenced by the high value of the shape-complementarity parameter (*Sc* = 0.79)^26^. The epitope is held tightly to the antibody by numerous contacts between a large patch of non-polar residues at both the CDRH3 of the Fab and the peptide, and by H-bonds between main-chain atoms of the antibody and critical residues Trp672 and Lys683 of the peptide (Supplementary Tables 3–5). The unprecedented detail of the structure of the extended region of 10E8ep (TMD residues ^684^LFIMIVG^690^) visualized for the first time in the complex with 10E8 reveals that residues Ile686 and Met687 are located in the proximity of the crucial residues Phe100a_HC_ and Trp100b_HC_ of the Fab (Fig. 2e). The combined buried surface area (BSA) of these four residues increased by 99 Å with respect to the complex between 10E8 and a peptide with a shortened C-terminal region (Supplementary Table 5). The enlarged interaction surface in the complex with the longer peptide is expected to favorably contribute to the high-affinity between antibody and antigen.

### Relevance of the extended region of 10E8ep for binding

**Figure 3:**
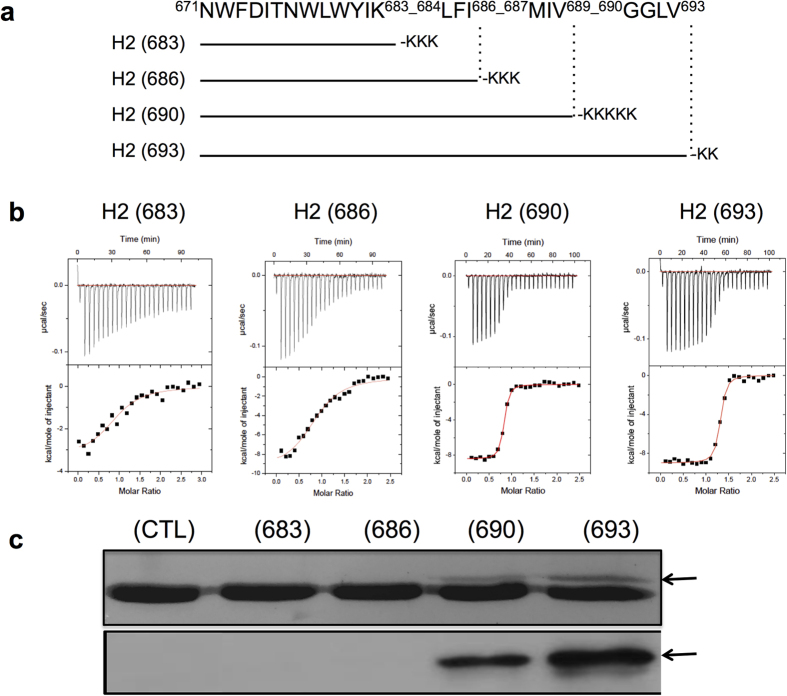
Binding of peptides spanning Helix-2 to Fab 10E8 in the presence of DPC. **(a)** Overview of the sequences of the H2 peptides displaying increasingly elongated C-termini. The four peptides differed in the number of consecutive helical turns following position 683. **(b)** Binding isotherms of the H2 peptide epitopes to Fab 10E8 examined by ITC. The thermodynamic parameters of binding are displayed in Table 1. (**c**) Photo-cross-linking between Fab 10E8 and the peptides. Protein bands were detected by Coomassie-blue staining or by western blot using 4E10 antibody (top and bottom panels, respectively). The arrows point to the position of the photo-cross-linked product. (CTL, control 10E8 Fab without peptide).

To establish the functional relevance of the interactions between 10E8 and the helical epitope extending beyond residue Lys683, the peptide epitopes H2(683), H2(686), H2(690) and H2(693) lacking the H1 region of MPER were generated by progressively adding helical turns to their C-terminus (Fig. 3a). Titration experiments with these peptides revealed different binding patterns depending on the extent of the elongation (Fig. 3b and Table 1). The affinity of the peptides increased in the order H2(683) < H2(686) ≪ H2(690) ≈ H2(693). Importantly, the affinity of the two longest H2 peptides H2(690) and H2(693) (10 ± 1.6 and 9.4 ± 2.0 nM, respectively) is comparable within error to that of the 10E8ep peptide (9.6 ± 1.0 nM), demonstrating that the H1 region of MPER does not contribute to high-affinity binding by this antibody. The thermodynamic signature is dominated by the large and favorable contribution of the enthalpy term, except for the shorter H2(683) peptide in which both entropy and enthalpy terms contribute similarly to binding. The lower affinity towards the H2(686) peptide appears to originate at the unfavorable contribution of the entropy term. We speculate that hydrophobic surfaces in this truncated helix are not properly oriented for effective binding. Perhaps the poly-Lys tags can distinctly affect 10E8 CDRH3 loop and/or micelle curvature in this shorter construct. Furthermore, an important question to be solved is whether the enthalpic energy coming from varying orientation of the longer 10E8ep, H2(686), H2(690) and H2(693) constructs relative to membrane is as important as to the direct contacts alone, which presumably predominate in the case of the shorter H2(683) version. Solving these issues will however require a comprehensive characterization of the solution structure of each peptide.

To obtain direct evidence about the non-polar contacts between the tip of the CDRH3 and residues downstream of position Lys683 of the peptide, we carried out photo-cross-linking experiments. The 10E8 Fab was modified with the UV-sensitive unnatural amino acid *p*-benzoylphenylalanine (*p*BPA)^27^,^28^, which was genetically encoded at position Trp100b_HC_ of the antibody (Fig. 3c and Supplementary Fig. 5). The Fab-peptide complexes were subjected to UV light and the formation of covalent adducts analyzed by SDS-PAGE. An additional band corresponding to cross-linked peptide and Fab HC was observed in samples containing Fab and H2(690) or H2(693), but not with H2(683) or H2(686) (Fig. 3c). The new band was absent in the Fab:peptide samples before irradiation (CTL lane) demonstrating it is specifically created by UV-mediated cross-linking. Thus, inclusion of the full MPER-N-TMD helix (H2) sequence enabled additional interactions with the tip of the 10E8 CDRH3, increasing significantly the affinity for the antibody.

### Recognition of the MPER-N-TMD helix at the membrane interface

**Figure 4:**
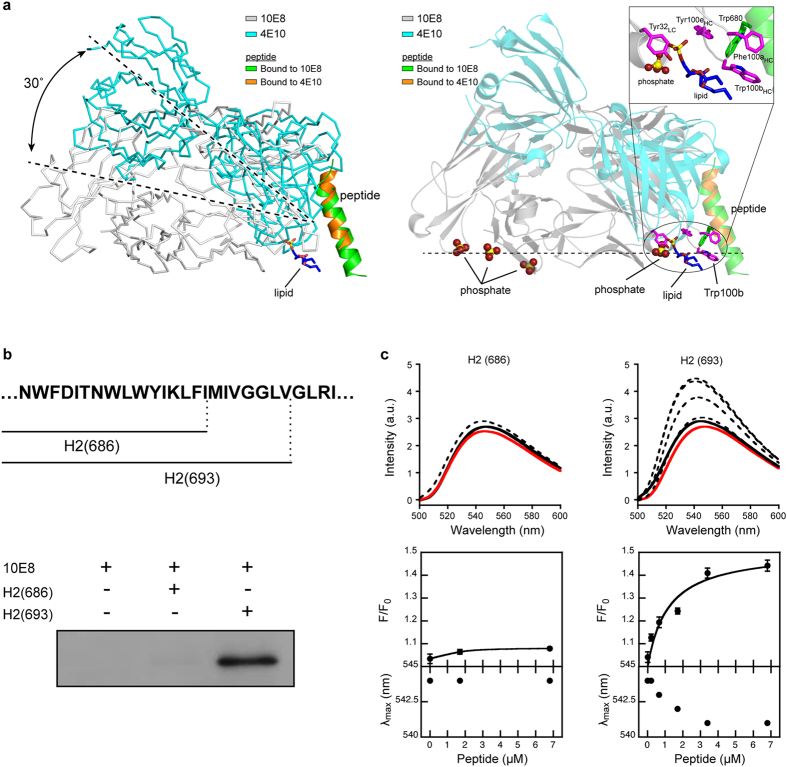
Binding to the membrane-inserted epitope. **(a)** Model of the recognition of the epitope by 10E8 and 4E10 Fab in membranes. Left: Relative orientation of 10E8 (gray) with respect to 4E10 (PDB entry code 2FX7, cyan) in membranes. The position of the peptide is such that occupies an oblique orientation as in Irimia *et al*.^16^. The position of 10E8 was calculated after superimposing residues 673-678 of the peptide bound in the structures with 10E8 or 4E10 (RMSD = 0.25 Å). The angle between the two Fab chains was calculated with CHIMERA. The lipid molecule bound to 4E10 was obtained from the structure (PDB entry 4XCN). Carbon, oxygen and phosphate atoms of the lipid are depicted with blue, red and yellow sticks, respectively. Right: Several phosphate ions (red and yellow) are bound to 10E8, suggesting a putative orientation of the antibody with respect to the surface of the viral membrane (represented with a dotted line). 10E8 (gray) and 4E10 (cyan) Fabs are shown in the same orientation as in the left panel. Residue Trp100b_HC_ of 10E8 and the molecule of lipid bound to 4E10 are depicted in magenta and blue, respectively. Inset: Close-up view of the circled region. **(b)** Photo-cross-linking of Fab 10E8 with peptides H(686) and H(693) inserted in membranes. Protein bands were detected by western-blot. Other experimental conditions are identical to those described in Fig. 3c. (**c**) Membrane insertion of the 10E8 CDRH3 apex probed by NBD fluorescence. Top panels: Spectra of NBD emission were measured upon incubation of NBD-Fab in solution (red traces), with liposomes devoid of peptide (black solid traces) or with LUV-peptide complexes that contained increasing amounts of H(686) or H(693) (black dotted traces in left and right panels, respectively). Bottom panels: Fractional changes (top) and maximum shifts (bottom) of NBD emission deduced from the spectra. The initial fluorescence (F_0_) was determined from NBD-Fab in buffer samples (red traces). Final fluorescence values (F) were measured after the addition of LUV-peptide complexes (black traces). Lipid concentration was fixed at 250 μM. Means ± S.D. of three independent experiments are represented. The lines correspond to the best fit of the experimental values to a hyperbolic function.

A previous report employing short lipids revealed the orientation of 4E10 Fab with respect to the plane of the membrane^16^. Because the broadly neutralizing antibodies 10E8 and 4E10 recognize approximately the same segment of MPER, we inferred the relative orientation between antibodies by simply superimposing the coordinates of the peptide in the crystal structures (RMSD = 0.25 Å) (Fig. 4a, left). The comparison revealed that the arrangement of 10E8 Fab is different from that of the 4E10 Fab, with each antibody binding a somewhat different face of the peptide; consequently, they approach the helix from different orientations with an estimated difference of 30°. According to this simple model, 10E8 would approach the epitope from a direction parallel to the plane of the membrane. This model predicts that the variable and constant regions of the 10E8 light chain interact, at least weakly, with the surface of the viral membrane. This hypothesis was reinforced by two separate observations from the crystal structure (Fig. 4a, right, and Supplementary Fig. 6). First, a phosphate ion bound to the CDRL1 of 10E8 (residues 28LC-32LC) occupies a similar position to that of a lipid bound to 4E10 (PDB entry code 4XBG). Supporting its adaptive role, this cognate site appears mutated at position 31LC with respect to the non-mutated germline ancestor inferred for 10E8^3^. And second, three phosphate ions, scattered across the constant domain of the light-chain, occupy positions consistent with the location of the headgroups of lipid molecules. Although the relevance of these sites is not demonstrated herein, this region of the antibody is expected to sit on the viral membrane when bound to Env^14^. Collectively, the model in Fig. 4a suggests that the Fab region of the 10E8 antibody approaches laterally to the MPER helix, the peptide being inserted obliquely into the lipid bilayer.

The orientation of 10E8 Fab with respect to the membrane places the CDRH3 tip immersed into the lipid bilayer. Hence, if the hydrophobic interactions with the C terminus of the peptide described above were relevant for the activity of the antibody they must occur inside the membrane (Fig. 4a, right). To test this prediction, we used Fabs incorporating *p*BPA, or Fabs labeled with the polarity-sensitive fluorescent probe NBD, and compared their interactions with the short H2(686) and long H2(693) peptides inserted in membranes (Fig. 4b & c). In this experiment, we first established conditions for the quantitative partitioning of the peptides into large unilamellar vesicles (LUVs) (Supplementary Fig. 7). Incubation of the LUV-peptide complexes with 10E8 Trp100b_HC_*p*BPA Fab, followed by irradiation with UV-light, generated Fab-peptide adducts in the presence of the long peptide H2(693) peptide, but not in the presence of the shorter one H2(686) (Fig. 4b). This experiment corroborated that the interactions observed in presence of DPC micelles (Fig. 3c) are also reproduced in the environment of biological membranes.

To determine the degree of penetration of the CDRH3 apex of Fab 10E8 into lipid membranes, Trp100b_HC_ was replaced by Cys, and modified chemically by attaching an NBD dye to its sulfhydryl group^29^,^30^. The fluorescence intensity of NBD increases and shifts to shorter wavelengths upon transferring from an aqueous solvent into the less polar environment of the bilayer (Supplementary Fig. 8). Thus, the extent of the interaction between the labeled NBD-Fab and the membrane was evaluated by comparing the emission of fluorescence of NBD before and after incubation with LUV-peptide complexes (Fig. 4c). The fluorescence spectrum of NBD did not change appreciably upon incubation of the labeled Fab with bare vesicles, or with vesicles containing the short H2(686) peptide (Fig. 4c). In contrast, incubation of Fab with vesicles containing the longer peptide H2(693) enhanced fluorescence emission, accompanied by a shift of the maximum towards lower wavelengths. Importantly, the changes described correlated with the density of H2(693) in the membrane (Fig. 4c), and were not observed with vesicles decorated with the H2(693) peptide bearing a double-Ala substitution for residues Trp672 and Phe673 (Supporting Fig. 8b); this result strengthens the conclusion that a change in polarity sensed by the Fab-probe is the consequence of specific binding to the H2(693) peptide. Collectively, the results in Fig. 4 suggest that recognition of the epitope in a membrane setting requires the interaction with the tip of the 10E8 CDRH3, an idea that is further reinforced by the data from the crystal complex (Fig. 2).

### Biological activity

**Figure 5:**
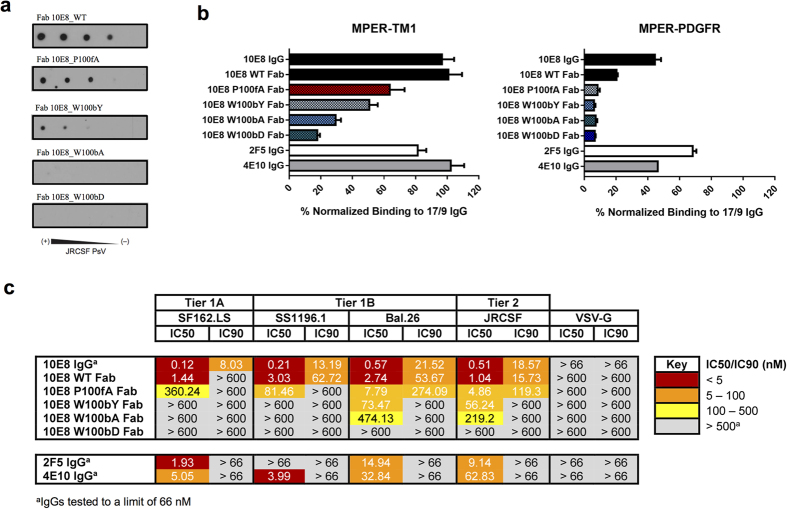
Effect of substitutions in CDRH3 on Fab binding to the 10E8 epitope in the context of the plama membrane and correlation with neutralizing activity. (**a**) JRCSF PsV recognition by 10E8 Fab and mutein Fabs in a dot-blot assay. Decreasing amounts of PsV (from left to right) were spotted onto nitrocellulose membranes and probed with 10E8 Fab and its muteins. (**b**) Cell lysates bearing recombinant MPER proteins (MPER-TM1: left; MPER-PDGFR: right) were probed with 20 nM IgG or Fab, as indicated, and binding signals were normalized to that of 20 nM 17/9 IgG, which binds to N-terminal HA tags on both constructs. (**c**) Neutralization profile of a panel of PsVs bearing HIV Env or that of vesicular stomatitis virus (negative control). IC_50_ and IC_90_ values for 2F5, 4E10 and 10E8 IgGs, 10E8 Fab and its muteins are shown.

We next evaluated binding for 10E8 in membrane-like environments in relation to its biological activity by producing 10E8 Fabs bearing substitutions in the critical CDRH3 loop (Table 2, Fig. 5 and Supplementary Fig. 9). Two separated Ala substitutions, namely Pro100f_HC_Ala, and Trp100b_HC_Ala, were selected based upon previous work indicating a lack of correlation between affinity for the peptide and neutralization potency^1^. In addition, Fab 10E8 muteins bearing conservative Trp100b_HC_Tyr and non-conservative Trp100b_HC_Asp substitutions were prepared to determine the role of the aromatic residue, Trp100b_HC_, in optimizing the Fab-antigen hydrophobic contact surface (Supplementary Table 5). After expression and purification, circular dichroism data indicated that the four muteins retained the overall structure of the unmodified WT Fab (Supplementary Fig. 10).

Titration of the muteins with peptide in solution (Supplementary Fig. 9 and Table 2) revealed diminished affinity for the 10E8ep peptide between 5- and 39-fold. The experimental order of affinities was WT > Trp100b_HC_Tyr > Pro100f_HC_Ala > Trp100b_HC_Ala ≈ Trp100b_HC_Asp. Examination of the thermodynamic parameters did not clarify the energetic mechanism behind the loss of affinity, although we note that in all cases the weaker binding was still governed by a favorable change of enthalpy (Δ*H* < 0 kcal mol^−1^).

The ranking of affinities changed when the 10E8 epitope was presented in the context of immobilized pseudovirus (PsV) particles (Fig. 5a). The dose-dependent binding of the Fabs to immobilized PsV particles diminished gradually for Pro100f_HC_Ala and Trp100b_HC_Tyr variants, and was undetectable for Trp100b_HC_Ala or Trp100b_HC_Asp variants. Thus, in the context of the viral surface the Pro100f_HC_Ala variant bound more efficiently to the 10E8 epitope than the conservative Trp100b_HC_Tyr one.

To determine if this effect was induced by membrane anchoring, the 10E8 epitope was next presented in the plasma membranes of cells expressing the fusion polypeptide, MPER-TM1, which comprises (i) an N-terminal HA tag, (ii) the MPER residues, and (iii) the TMD residues of gp41 followed by 27-AA of the gp41 cytoplasmic domain (Fig. 5b, left)^31^. Previous studies of MPER-TM1 revealed that the 4E10 epitope is well exposed for antibody binding when constrained by the gp41 TMD; in contrast, 4E10 binds weakly to a construct in which the gp41 MPER-TMD junction is disrupted by fusion to another receptor’s 20-AA membrane-proximal external region and TMD (MPER-PDGFR). In this previous work, the C-terminal helix of the MPER was envisioned as being continuous with the gp41 TMD helix; this arrangement was thought to fully expose the MPER for 4E10 docking^31^.

Figure 5b shows that both 4E10 and 10E8 IgGs bind tightly to the MPER-TM1 in the context of the plasma membrane. These antibodies engaged with the MPER-TM1 construct as effectively as the 17/9 IgG, which binds to the solvent-exposed HA epitope and served to normalize expression levels^31^. A comparable degree of binding was observed for the WT 10E8 Fab, thereby sustaining side-by-side comparative assessment of its CDRH3 muteins. The binding pattern of substituted Fabs to MPER-TM1 coincided with that previously observed in PsVs. In contrast, the MPER-PDGFR construct was a poor ligand for both the 4E10 and 10E8 IgGs, and for the 10E8 Fab and its muteins, whereas, 2F5, whose epitope is on the N-terminal sub-region of the MPER, was marginally affected (Fig. 5b, right). None of the mutant Fabs bound to the MPER-PDGFR in the membrane context. These results reflect the ability of the gp41 TMD residues to affect the positioning of 10E8 epitope at the membrane interface, and demonstrate the requirement of the hydrophobic CDRH3 tip for the occurrence of the recognition process.

The differences observed in binding of the 10E8 Fab and its muteins to PsVs or MPER-TM1 were mirrored by differences in their neutralization potencies (Fig. 5c). When characterizing the neutralization breadth and potency of antibodies, it is common to choose a number of pseudovirus categories (tiers), with varying sensitivity to Ab-mediated neutralization^32^. In our studies, we selected HIV-1 Env variants that were highly sensitive to Ab-mediated neutralization (SF162.LS; Tier 1 A), those that demonstrate above-average sensititivity (SS1196.1 and Bal.26; Tier 1B), and one that demonstrated low-sensitivity (JRCSF; Tier 2). The neutralization assays confirmed the previously reported detrimental effects induced by the Pro100f_HC_Ala and Trp100b_HC_Ala substitutions^1^, which rendered a less potent, but still functional Fab, and an almost non-neutralizing variant, respectively. Moreover, in comparison to the Trp100b_HC_Ala mutein, the Fab bearing the conservative Trp100b_HC_Tyr substitution gained neutralization potency. However, the activity of the Trp100b_HC_Tyr variant was lower than that of the Pro100f_HC_Ala variant. Finally, the non-conservative Trp100b_HC_Asp substitution rendered a Fab devoid of all activity.

## Discussion

In this work, we have provided new insights into the molecular mechanism linking the binding of the helical epitope at the membrane interface with the biological function of 10E8. The chemically heterogeneous membrane interface region allows multiple types of non-covalent lipid-peptide interactions^33^,^34^. Importantly, aromatic residues, and particularly Trp, have a tendency to preferentially locate within this region^35^,^36^. Thus, interactions with lipids and the spatial arrangement of favorably inserted aromatic residues may influence the orientation of MPER epitopes at the membrane surface, the mechanism of antibody docking, or both. Previous NMR studies^13^ suggested that preservation of the continuous MPER-N-TMD helix inserted in the interface could be critical for high affinity binding to epitopes of the C-terminal MPER sub-region (Fig. 1a). The 10E8ep peptide was designed to preserve this element while including all residues that were in contact with the 10E8 antibody in the first reported structure of the Fab-peptide complex (Fig. 1b). Elucidation of the NMR structure in a membrane-mimetic environment corroborated the conformational flexibility at position ^671^NW^672^ predicted for 10E8ep, and the existence of a built-in, continuous C-terminal helix (Fig. 1c). This structure was bound with high affinity by the 10E8 Fab forming a stable complex in the presence of DPC micelles (Fig. 1d & e).

The level of detail attained in the structure of the complex between the peptide 10E8ep and the complex in the presence of DPC supports previously reported data regarding the binding of peptides systematically substituted with Ala^1^, recent cryo-EM reconstructions^14^, and more recent crystallographic studies^10^, indicating that 10E8 binding affinity is primarily mediated by its mode of recognition of the shorter ^671^NWFDITNWLWYIK^683^ sequence (Fig. 2). However, structure resolution of the complete helix ^671^NWFDITNWLWYIKLFIMIVG^690^ in complex with Fab adds three important factors to our understanding of the 10E8 epitope.

First, we propose the absence of a kink interrupting the MPER helix at position Lys683 and the oblique insertion of the whole structural element into the membrane. Thus, our crystallographic data (Figs 2 and 4 and Supplementary Fig. 6) are consistent with recent models suggesting that the main axis of the uninterrupted helix of the epitope forms an oblique angle with respect to the membrane plane^13^,^16^, with some intermolecular contacts made by the anti-MPER Fabs occurring at the vertex, after engaging with the helix surface facing the membrane^14^,^16^. The absence of a kink at position Lys683 is also supported by the recent structural resolution of a trimeric TMD sequence embedded in lipid bicelles^37^. A widely assumed alternative model suggests that the MPER helix inserts in parallel to the membrane interface and kinks at position Lys683 to allow the perpendicular insertion of the TMD helix starting at position Leu684^17^,^19^,^20^,^22^,^38^. Accordingly, it has been proposed that anti-MPER antibodies approach from above the membrane-inserted MPER epitopes^10^,^18^,^20^,^21^,^38^,^39^,^40^. We note here that, although our structural data and the cryo-EM studies by Lee *et al*.^14^ complement each other, suggesting that 10E8 binds to the Env glycoprotein in a CD4/bound-like state, they do not rule out the possible existence of a native Env state including the H1 section of the MPER embedded into the membrane-interface. Such pre-fusion state could also be recognized by anti-MPER antibodies, as it is assumed by some previous models^19^,^21^,^39^. In the context of that recognition mechanism, a different 10E8 surface would contact the membrane interface, as suggested by recent mutagenesis studies^10^.

Second, our data clearly suggest the active participation of residues beyond Lys683 in the interaction with Fab in the membrane environment. Titration, photo-cross-linking data, and structural analysis revealed the involvement of residues Ile686 and Met687 in establishing non-polar contacts with the CDRH3 apex residue, Trp100b_HC_; the interaction surface of this residue is doubled in the presence of the long peptide (Figs 2e and 3, and Supporting Table 5). Collectively, the identification of several phosphate groups on the surface of the antibody in the crystal structure (Fig. 4a and Supporting Fig. 6), and the capacity of the Fab to recognize the membrane-anchored peptide (Fig. 4b & c) suggest that key Fab-peptide interactions required for the effective neutralization occur within the membrane interface. It appears that the maximum binding potential of 10E8 emerges from the simultaneous interactions of Trp100b_HC_ with TMD residues Ile686 and Met687 and phospholipids (Figs 2e & 4a). We speculate that an optimal orientation attained in the membrane milieu could enable sulfur/π interactions between residues Met-687 and Trp100b_HC_^41^.

**Figure 6:**
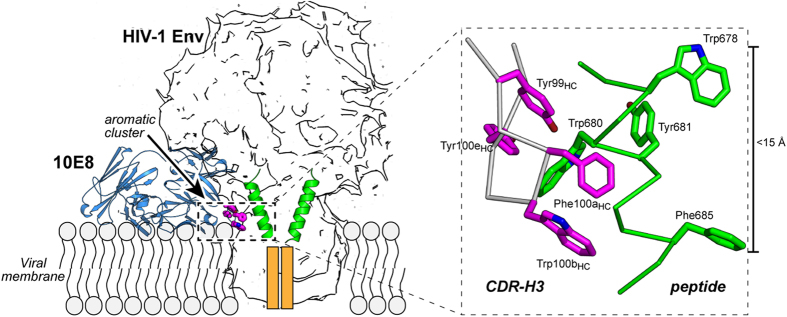
Model for 10E8 epitope binding at the viral membrane interface. A silhouette model of the trimer of Env from Lee *et al*.^14^ was employed to illustrate the figure. To facilitate the understanding of the model, only two MPER epitopes (green helices) are shown. The antibody is shown in blue, and the critical aromatic cluster depicted as magenta sticks. Orange boxes represent deeper segments of the TMD. Inset: close-up view of the aromatic residue cluster stabilizing the MPER-N-TMD helix at the membrane interface. Residues contributed by Fab and gp41 are depicted in magenta and green, respectively.

And third, the mutational analysis of the 10E8 CDRH3 region indicates that preservation of such interactions directly correlates with the neutralizing activity of the antibody (Table 2 and Fig. 5). Thus, the binding of the 10E8 Fab and its muteins to Env-bearing PsVs or to the MPER-TM1 polypeptide in the context of the plasma membrane reflected with remarkable accuracy their neutralizing activity (Fig. 5). The model displayed in Fig. 6, which combines the high-resolution structural information described in this work, with that derived from cryo-EM reconstructions^14^, provides a putative mechanism to explain that correlation.

The model proposes that high-affinity binding to the 10E8 epitope at the membrane interface requires engagement with the N-terminal core-epitope residues, and establishment of a firm grip along the full MPER-N-TMD helix, including the interaction between Trp100b_HC_ and TMD residues, Ile686 and Met687. Assuming that a recently reported trimeric structure might represent the organization of the TMD in the native Env^37^, full achievement of that interaction implies that the 10E8 antibody engages the epitope after rotation and uplifting of the TMD helix. Thus, the stringent dependence on the aromatic nature of the Trp100b_HC_ residue for biological activity might be due to the fact that, to establish an effective grip, the CDRH3 apex must submerge in the membrane interface^36^, and establish at the deepest level tight contacts with the C terminus of the MPER-N-TMD helix surrounded by membrane phospholipids. Holding the helices in the uplifted position observed in the 10E8-bound form of the Env trimer^14^ would be energetically favored by the network of interfacial aromatic residues that are contributed upon binding by both the Fab and the MPER-N-TMD helix (Fig. 6, inset). We speculate that the stabilization of monomeric helices in this state might be incompatible with the progression of Env-mediated fusion explaining, at least in part, the mechanism by which 10E8 blocks viral fusion.

In summary, we conclude that, for effective neutralization, the antibody must combine the specific binding to MPER residues transiently exposed to solvent at the membrane surface, with establishment of downstream interactions with the TMD at the deepest levels of the membrane interface. Thus, this model may guide the future design of immunogens to more effectively elicit 10E8-like antibodies.

## Materials and Methods

Peptides were synthesized in a C-terminal carboxamide form by solid-phase methods using Fmoc chemistry, purified by reversed- phase HPLC, and characterized by matrix-assisted time-of-flight (MALDI-TOF) mass spectrometry (purity >95%). Peptides were routinely dissolved in dimethylsulfoxide (DMSO, spectroscopy grade) and their concentration determined by the bicinchoninic acid microassay (Pierce). Solubilization Lys-tags were added to the synthetic sequences to attain the solubility required for performing NMR and ITC studies. Anti-HIV-1 gp41 antibody 4E10 (from Dr. Hermann Katinger) and pNL4-3.Luc.R-.E- (from Dr. Nathaniel Landau) were obtained through the NIH AIDS Reagent Program, Division of AIDS, NIAID, NIH. MAb 17/9 was kindly provided by R. Stanfield and I. Wilson (The Scripps Research Institute, La Jolla, CA). Secondary antibodies, goat (anti-human kappa light chain)-horseradish peroxidase (HRP) conjugate, and goat (anti-human antibody)-HRP conjugate were purchased from Life Technologies. Experimental procedures described in^42^ were followed for the production and purification of Fabs. Vector pEVOL, encoding a Tyr-tRNA synthetase suitable for the incorporation of the photoreactive amino-acid, p-benzoylphenylalanine (*p*BPA), was a gift from Prof. P. G. Schultz (The Scripps Research Institute), and, *p*BPA was purchased from Bachem. 4-Chloro-7-Nitrobenz-2-Oxa-1,3-Diazole (NBD) was from Molecular Probes.

Recording of NMR spectra, assignments and structure calculations were carried out as previously reported^13^ (see Supplementary Methods). Isothermal titration calorimetry (ITC) experiments were performed with a VP-ITC microcalorimeter (MicroCal, Northampton, MA) at 25 °C. Prior to the experiment, proteins were dialyzed overnight at 4 °C against 10 mM sodium phosphate (pH 7.5), 150 mM NaCl, and 10% (v/v) glycerol. Samples containing protein and peptide solubilized in dialysis buffer were supplemented with 5 mM DPC and degassed immediately before each measurement. Fab10E8 (3 μM) was titrated with peptide (40 μM). The volume of each injection was 10 μL. Peptide dilution heat was subtracted for data analysis. The binding isotherms were fitted to a one-site binding model using the program ORIGIN 7.0 (MicroCal, Northampton, MA). The fitting procedure yields the stoichiometry (*n*), the binding constant (*K*_*D*_) and the enthalpy (*ΔH°*) of the binding reaction. For differential scanning calorimetry (DSC) determinations, heat capacity was measured using a VP-DSC scanning microcalorimeter (MicroCal, Northampton, MA). Temperature scans were performed in a 10 mM sodium phosphate (pH 7.5), 150 mM NaCl, and 10% glycerol buffer supplemented with 5 mM DPC. Samples of 10E8 (10 μM) with and without peptide (15 μM) were heated from 30 to 90 °C at a rate of 1 °C min^−1^. The ORIGIN software package (MicroCal) was used for data collection and analysis. The buffer baseline was subtracted from the sample raw data, normalized by protein concentration, and fitted with a two-state thermal transition model to obtain thermodynamic parameters.

Crystals of the complex between 10E8 Fab (3 mg L^−1^) and the peptide 10E8ep added at a molar ratio of ~1:1.5 (Fab:peptide) in the presence of 2.5 mM DPC were grown in a solution of 50% (v/v) 2-methyl-2,4-pentanediol, 200 mM phosphate, and 100 mM TRIS (pH 8.5) at 20 °C. A single protein crystal was harvested and stored in liquid nitrogen until data collection at the synchrotron. Data collection, processing and structure calculations are described in the Supplementary Methods.

For the photo-cross-linking experiments, an amber codon specific for an engineered tRNA that translates the unnatural amino acid, *p*BPA, was encoded in the DNA sequence of the heavy chain of the 10E8 Fab. Procedures to express a 10E8 Fab mutein bearing *p*BPA instead of Trp at position 100b_HC_ were adapted from previous reports^27^,^28^. Synthesis of Fab and the engineered tRNA was induced with 0.4 mM isopropyl-D-thiogalactopyranoside (IPTG) and 4% (w/v) arabinose, respectively, in LB medium supplemented with 0.2 mg/L *p*BPA. For the photo-cross-linking experiment, samples containing Fab bearing the Trp100b_HC_pBPA substitution at 1.5 μM and peptides at 10 μM were irradiated with UV light at 365 nm for 20 min at 4 °C using a UVP B-100AP lamp. Peptide binding in solution and in lipid membranes was accomplished in the presence of 5 mM of DPC or 1.5 mM of large unilamelar vesicles (LUVs), respectively. The LUVs were composed of 1,2-dioleoyl-*sn*-glycero-3-phosphatidylcholine,1,2-dioleoyl-*sn*-glycero-3 phosphatidylethanolamine 1,2-dioleoyl-*sn*-glycero-3- phosphatidylserine, and egg sphingomyelin (27:29:14:30, mole ratio), and were produced by extrusion through two stacked polycarbonate membranes with a nominal pore size of 0.1 μm (Nuclepore Inc.). Fab heavy-chain-peptide adducts were separated by SDS-PAGE and stained with Coomassie blue. Alternatively, they were identified by western blot using a sandwich comprising a goat (anti-human Fab) antibody (Sigma) and a mouse (anti-goat) antibody-HRP conjugate (Santa Cruz). Photo-cross-linked products were monitored using an LAS-4000 image analyzer (GE Healthcare).

Labeling with the polarity-sensitive NBD probe was performed as described^29^,^30^. In brief, a cysteine-substituted Fab derivative (Trp100b_HC_Cys) was first generated by site-directed mutagenesis, then produced and modified with a sulfhydryl-specific iodoacetamide derivative of NBD. Fluorescence-emission spectra were recorded with the excitation wavelength fixed at 470 nm. An emission spectrum of a sample lacking the fluorophore was subtracted from the spectrum of the equivalent sample containing the fluorophore. Fluorescence spectra of NBD were obtained upon incubation of NBD-labeled Fab (0.5 μM) with liposomes (total lipid concentration 250 μM) containing increasing amounts of peptide H(686) (1.7 and 6.8 μM) or H(693) (0.2, 0.65, 1.7, 3.4, and 6.8 μM).

293-T cells expressing DNA constructs encoding the MPER-TM1 or the MPER-PDGFR polypeptide were lysed and used in an enzyme-linked immunosorbent assay (ELISA) as described in (29). Pseudoviruses (PsVs) bearing cloned HIV-1 Envs were used in a luciferase-based neutralization assay following our previously described protocols^31^,^42^ (see Supplementary Methods for a detailed description).

Dot blots were performed as described^43^. In brief, decreasing amounts of PsVs were spotted onto Hybond C nitrocellulose (GE Healthcare). The nitrocellulose was then blocked with 5% fat-free milk in PBS (Blocking Buffer) for 1 h and incubated for 1 more hour with antibodies (0.2 μg/ml) in Blocking Buffer at room temperature. The membranes were washed 3 times, 10 min each with PBS. Filters were developed using horseradish peroxidase-conjugated antibody.

## Additional Information

**Accession Codes**: Coordinates for 10E8ep structures were deposited in the Protein Data Bank under the following entries: 2NCS (in DPC) and 2NCT (in HFIP). 1 H and 13C chemical shifts were deposited at BioMagResBank with accession codes 26034 (in DPC) and 26035 (in HFIP). The coordinates and structure factors for the complex between the recombinant Fab 10E8 and the peptide 10E8ep have been deposited in the Protein Data Bank under accession code 5GHW

**How to cite this article**: Rujas, E. *et al*. Structural basis for broad neutralization of HIV-1 through the molecular recognition of 10E8 helical epitope at the membrane interface. *Sci. Rep.* **6**, 38177; doi: 10.1038/srep38177 (2016).

**Publisher's note:** Springer Nature remains neutral with regard to jurisdictional claims in published maps and institutional affiliations.

## Supplementary Material

Supplementary Information

## Figures and Tables

**Table 1:** Thermodynamic parameters of binding of Fab 10E8 to the peptide 10E8ep and peptides based on Helix 2.

Peptide	*K*_*D*_(nM)	Δ*G°* (kcal mol^−1^)	Δ*H°* (kcal mol^−1^)	*−T*Δ*S°* (kcal mol^−1^)^a^	*n*^b^
10E8ep	9.6 ± 1.0	−10.9 ± 0.1	−9.6 ± 0.1	−1.3 ± 0.2	1.0 ± 0.1
H2 (683)	746 ± 307	−8.3 ± 0.3	−3.3 ± 0.3	−5.0 ± 0.6	0.9 ± 0.1
H2 (686)	369 ± 84	−8.7 ± 0.1	−9.6 ± 0.5	0.9 ± 0.6	0.9 ± 0.1
H2 (690)	10 ± 1.6	−10.9 ± 0.1	−8.4 ± 0.1	−2.5 ± 0.2	0.8 ± 0.1
H2 (693)	9.4 ± 2.0	−10.9 ± 0.1	−9.0 ± 0.1	−1.9 ± 0.2	1.3 ± 0.1

^a^Temperature was 298 °K.

^b^***n*** refers to the molar ratio peptide/protein.

**Table 2:** Thermodynamic parameters of binding of Fab 10E8 CDRH3 muteins to the peptide 10E8ep.

*Antibody*	*K*_*D*_(nM)	Δ*G°* (kcal mol^−1^)	Δ*H°* (kcal mol^−1^)	*−T*Δ*S°* (kcal mol^−1^)^a^	*n*^b^
WT	9.6 ± 1.0	−10.9 ± 0.1	−9.6 ± 0.1	−1.3 ± 0.2	1.0 ± 0.1
Pro100f_HC_Ala	161 ± 22	−9.2 ± 0.1	−11.1 ± 0.2	1.9 ± 0.3	0.8 ± 0.1
Trp100b_HC_Tyr	52 ± 12	−9.9 ± 0.1	−8.9 ± 0.1	−1.0 ± 0.2	1.1 ± 0.1
Trp100b_HC_Ala	361 ± 82	−8.8 ± 0.1	−10.0 ± 0.5	1.2 ± 0.6	1.0 ± 0.1
Trp100b_HC_Asp	375 ± 66	−8.7 ± 0.1	−6.5 ± 0.3	−2.2 ± 0.4	1.0 ± 0.1

^a^Temperature was 298 K.

^b^***n*** refers to the molar ratio peptide/protein.
