# Supplementary material for: Structural basis for broad neutralization of HIV-1 through the molecular recognition of 10E8 helical epitope at the membrane interface
Source: Sci Rep. 2016 Dec 1;6:38177. doi: 10.1038/srep38177 (PMC5131266; doi:10.1038/srep38177)
Supplement: Supplementary Information [file srep38177-s1.pdf]

Supplementary Information

Structural basis for broad neutralization of HIV-1 through the molecular recognition of 10E8 helical epitope at the membrane interface.

Edurne Rujas[1,2], Jose M. M. Caaveiro[2,3*], Angélica Partida-Hanon[4], Naveed Gulzar[5], Koldo Morante[2,3], Beatriz Apellániz[1], Miguel García-Porras[1], Marta Bruix[4], Kouhei Tsumoto[2,3], Jamie K. Scott[5,6], M. Ángeles Jiménez[4], and José L. Nieva[1*]

[1]Biophysics Unit (CSIC, UPV/EHU) and Department of Biochemistry and Molecular Biology, University of the Basque Country, P.O. Box 644, 48080 Bilbao, Spain.

[2]Department of Bioengineering, Graduate School of Engineering, The University of Tokyo, Bunkyo-ku, Tokyo, Japan.

[3]Institute of Medical Science, The University of Tokyo, Tokyo, Japan.

[4]Institute of Physical Chemistry "Rocasolano" (IQFR-CSIC), Serrano 119, E-28006 Madrid, Spain

[5]Department of Molecular Biology and Biochemistry, Simon Fraser University, Burnaby, Canada.

[6]Faculty of Health Sciences, Simon Fraser University, Burnaby, Canada.

Supplementary Methods

Recording of NMR spectra

NMR samples were prepared by dissolving the lyophilized peptides in 0.5 mL of 2 mM HEPES buffer at pH 7.0 in H_2O/D_2O (9:1 ratio by volume) or in D_2O and containing either 25 % (v/v) 1,1,1,3,3,3-hexafluoro-2-propanol (HFIP-D2, 98%; Cambridge Isotopes Lab) or 20 mM deuterated dodecylphosphocholine (DPC-D38, 98%; Cambridge Isotopes Lab). Peptide concentrations were ~0.5 mM. pH was measured with a glass micro electrode, not corrected for isotope effects and adjusted by adding minimal amounts of NaOD or DCl. All samples contained sodium 2,2-dimethyl-2-silapentane-5-sulfonate (DSS) as internal reference for ^{1}H chemical shifts. Temperature of the NMR probe was calibrated using a methanol sample. ^{13}C δ-values were indirectly referenced by using the IUPAC-IUB recommended ^{1}H/^{13}C chemical shift ratio (0.25144953; [1]).

NMR spectra were recorded on Bruker Avance-600 or Avance-800 spectrometers, both equipped with a cryoprobe. 2D phase-sensitive two-dimensional correlated spectroscopy (COSY), total correlated spectroscopy (TOCSY), and nuclear Overhauser enhancement spectroscopy (NOESY) spectra were acquired by standard techniques using presaturation of the water signal and the time-proportional phase incrementation mode, as previously reported [2]. NOESY mixing times were 150 ms, and TOCSY spectra were recorded using 60 ms DIPSI2 with z filter spin-lock sequence. ^{1}H-^{13}C heteronuclear single quantum coherence (HSQC) spectra were acquired at ^{13}C natural abundance using standard pulse sequences [2]. Acquisition data matrices were defined by 2048 x 512 points in t_2 and t_1, respectively. Data were processed using the standard TOPSPIN program (Bruker Biospin, Karlruhe, Germany). The 2D data matrix was multiplied by either a square-sine-bell or a sine-bell window function with the corresponding shift optimised for every spectrum and zero-filled to a 2K x 1K complex matrix prior to Fourier transformation. Baseline correction was applied in both dimensions.

NMR spectra assignment

^{1}H NMR signals of peptides in each solvent conditions were assigned using the standard sequential assignment strategy [3] with the help of the SPARKY software (T. D. Goddard and D. G. Kneller, SPARKY 3, University of California, San Francisco). The ^{13}C resonances were identified on the basis of the correlations between the protons and the bound carbon atoms present in the ^{1}H-^{13}C-HSQC spectra.

Structure calculation

Structures for peptide 10E8ep in DPC micelles and in 25 % HFIP were calculated from distance and dihedral angle constraints derived from NMR parameters using the standard iterative procedure for automatic NOE assignment of the program CYANA 2.1 [4], which consists of seven cycles of combined automated NOE assignment and structure calculation of 100 conformers per cycle [5]. Distance constraints were obtained from the cross-peaks present in 150 ms 2D [^{1}H-^{1}H]-NOESY spectra, which were integrated using the standard SPARKY integration sub-routine (T. D. Goddard and D. G. Kneller, SPARKY 3, University of California, San Francisco), and the dihedral angle restraints for ϕ and ψ angles were derived from $^{1}H_{\alpha}$, $^{13}C_{\alpha}$ and $^{13}C_{\beta}$ chemical shifts

using the TALOS-N webserver (Yang Shen, and Ad Bax, J. Biomol. NMR, 56, 227-241, 2013; http://spin.niddk.nih.gov/bax/nmrserver/talosn/). Since no ambiguous constraints were found upon examination of the list of distance constraints resulting from the last automatic cycle, the 20 lowest target function structures of this cycle were selected as the final structure ensembles. The quality of these structures was assessed using PROCHECK/NMR [6] as implemented at the Protein Structure Validation Suite server (PSVS server: http://psvs-1_4-dev.nesg.org/). The structural ensembles calculated for 10E8ep have been deposited at the PDB data bank with accession codes 2NCT (in HFIP) and 2NCS (in DPC). The structural statistics data for these structures are provided in Table ST1. The structures were visualized and examined using the programs MOLMOL [7].

X-ray crystallography

Diffraction data were collected in beamline AR-NW12A at the Photon Factory (Tsukuba, Japan) under cryogenic conditions (100 K) at a wavelength of 1.000 Å. A combination of long cell-unit (z axis was 254 Å) and high mosaicity (1.3°) limited the maximum resolution at which the data was collected to 2.4 Å. Diffraction data were indexed, integrated with MOSFLM [8], and scaled with AIMLESS [9]. The structure was determined by the method of molecular replacement using a previous crystal structure of 10E8 (PDB entry code 4G6F) [10] with PHASER [11]. Refinement was performed with COOT [12] and REFMAC5 [13]. Structural validation was performed with COOT and with PROCHECK [14]. There are no Ramachandran outliers to report. Data collection and refinement statistics are summarized in Table 2. BSA and polar interactions were identified with the PISA server [15], and the *Sc* parameter was calculated with SC of the CCP4 suite [16]. The elbow angles between the variable and constant regions of 10E8 in different crystal structures were calculated with the program RBOW [17]. The relative angle between 4E10 (PDB entry code 4XBG) [18] and 10E8, as well as other calculations were carried out with CHIMERA [19].

Cell lysate production

Following our previously described protocol [20], 293T cells were transiently transfected with 1 µg plasmid DNA encoding recombinant MPER proteins, MPER-TM1 and MPER-PGDFR (described below and in ref. [20]), using the XtremeGENE 9 transfection reagent (Roche, Basel, Switzerland), according to the manufacturer's instructions, at a ratio of 1:6 (i.e., µg DNA to µL transfection reagent). Cells were cultured in 6-well plates (Sarstedt, Numbrecht, Germany) in Dulbecco's modified Eagle's medium (DMEM; Life Technologies, location) supplemented with 10% (v/v) fetal calf serum (FCS; Life Technologies) and 1 mM L-glutamine (Life Technologies) at 37 °C and 5% CO_2. After 48 hours, the cells were washed four times in phosphate-buffered saline (Life Technologies), and recovered from the plate with 1 mM Na_2EDTA-NaOH, pH 8.0 (Bioshop, Burlington, Canada). Cells were pelleted by centrifugation for 5 min at 350 x *g*, resuspended in 200 µL lysis buffer comprising a protease-inhibitor cocktail (cOmplete ULTRA Tablets, mini, EDTA-Free; Roche) diluted 1:12 in (250 mM sucrose and 0.5 mM Na_2EDTA-NaOH, pH 8.0). Cell lysates were produced by 30 passages through a 22-gauge needle followed by two 15-s pulses with a Virsonic sonicator (VirTis, Gardiner, NY). Total protein content was quantified using the Bradford reagent (Bio-Rad Protein Assay, BioRad, Mississauga, Canada) following the manufacturer's instructions; lysates were stored at -80 °C.

Enzyme-linked immunosorbent assays (ELISAs)

Wells of high-binding microtiter plates (Corning Inc., Corning, NY) were coated overnight at 4 °C with 35 µL/well of one of the following diluted in Tris-buffered saline (TBS; 50 mM Tris-HCl, pH 7.5, 150 mM NaCl): (i) cell lysate (the equivalent of 10 µg protein), (ii) r-gp41 (50 ng), (iii) biotinylated $MPER_{667-683}$ peptide (400 ng), or (iv) TBS containing 2% (w/v) bovine serum albumin (TBS-BSA; Sigma-Aldrich). Wells were blocked for 1 h at room temperature (RT) with TBS-BSA, then washed twice with TBS containing 0.1% (v/v) Tween-20 (TBS-T), once with TBS, then incubated for 2 h at RT with the indicated dilutions of 4E10 IgG1, 17/9 IgG1 or one of the 4E10 Fabs (WT, WDWD, ΔLoop) diluted in TBS-T containing 5% (w/v) non-fat dried milk (5% NFDM; Bio-Rad). After five washes with TBS-T followed by one with TBS, bound 4E10 IgG or 4E10 Fabs were detected with goat (anti-human kappa light chain IgG)-HRP, and bound 17/9 IgG1 with goat (anti-murine IgG)-HRP, diluted 1:500 and 1:1,000, respectively, in 5% NFDM. After 1 h incubation at RT, plates were washed five times in TBS-T and once in TBS. Bound HRP was detected using 400 ng/ml 2'2'-azino-bis-3-ethylbenzthiazoline-6-sulfonic acid (ABTS; Sigma-Aldrich, St. Louis, MO) in developer solution comprising a 1:1.5 ratio of citrate (0.1 M, pH 2.4) and phosphate (0.2 M, pH 9.2) buffers, along with 0.03% (v/v) H_2O_2. Absorbance was measured at 405 - 490 nm, using a Tecan Infinite M200 Pro microplate reader (Tecan, Mannedorf, Switzerland).

HIV-1 envelope neutralization assays

Neutralization assays were performed using a single-round infection assay with envelope (Env)-pseudotyped virus (PsV) as described previously [21]. Briefly, PsVs were produced by co-transfection of 293T cells with (i) an Env-negative HIV genomic vector that carries a firefly luciferase reporter gene (pNL4.3.Luc.R.E-), and (ii) a plasmid carrying an HIV envelope gene expression cassette. 293T cells (2 x 106 cells) were co-transfected with plasmid DNA at ratios of 1:3 to 1:20 µg pNL4.3.Luc.R.E- plasmid DNA to µg HIV Env+ plasmid DNA and 54 µg polyethylenimine (PEI; Polysciences, Warrington, PA). Plasmids encoding Env from HIV-1 isolates and controls include: SF162.LS, SS1196.1 clone 1 (SVPB9), Bal.26, or JR-CSF, or with a plasmid expressing the Env from vesicular stomatitis virus (to produce a negative-control PsV). PsVs were harvested from cell-free supernatants 72 h post-transfection, passed through a 0.45 µm-filter, diluted 1:10 in 10X phosphate-buffered saline (Life Technologies), aliquotted, and stored at -80 °C.

PsV infection is measured using a luciferase-based assay in TZM-bl cells, with neutralization being measured as a reduction in luciferase activity following single-round infection. TZM-bl cells were seeded overnight (10,000/well) in flat-bottom 96-well plates (Corning) in a total volume of 200 µL DMEM supplemented with 10% heat-inactivated FCS and 1 mM L-glutamine (DMEM-ΔFCS). Three-fold serial dilutions of the appropriate MAb or Fab concentration were prepared in DMEM-ΔFCS in a total volume of 150 µL, and added in triplicate to the wells of a separate round-bottom 96-well plate (Corning). PsVs were added at 200 TCID50/well in a total volume of 50 µL DMEM-ΔFCS supplemented with 25 µg/mL diethylaminoethyl-dextran hydrochloride (DEAE-Dextran; Sigma-Aldrich), and plates were incubated for 1 h at 37 °C and

5% CO_2. Following incubation, media was removed from wells of the TZM-bl-seeded 96-well plates, and PsV-MAb/Fab samples were transferred to the plates. Control wells comprising TZM-bl cells only (cell control), and virus only (virus control) were also included. After a 72-h incubation, media was removed from wells, 50 µL Glo lysis buffer (Promega, Madison, WI) was added to each well, and lysis was allowed to proceed at RT with gentle rocking for five min. One-Glo luciferase substrate (Promega) (50 µL/well) was added and incubated for five min at RT. Samples were transferred (50 µL/sample) to 96-well, white solid plates (Thermo Fisher Scientific, Waltham, MA) and luciferase activity was measured using a Tecan Infinite M200 Pro multi-mode plate reader (Tecan). All values are reported as relative luminescence units (RLU), and neutralization was calculated with respect to cell- and virus-control wells using the following formula: 1 - [(RLUsample wells – RLUcells-only wells) / (RLUvirus-only wells – RLUcells-only wells)] x 100. The 50% inhibitory dose (ID50) concentration is calculated as the antibody/Fab concentration producing a 50% reduction in RLU compared to the level in the virus-control wells after subtraction of cell-control RLU.

Supplementary Figures

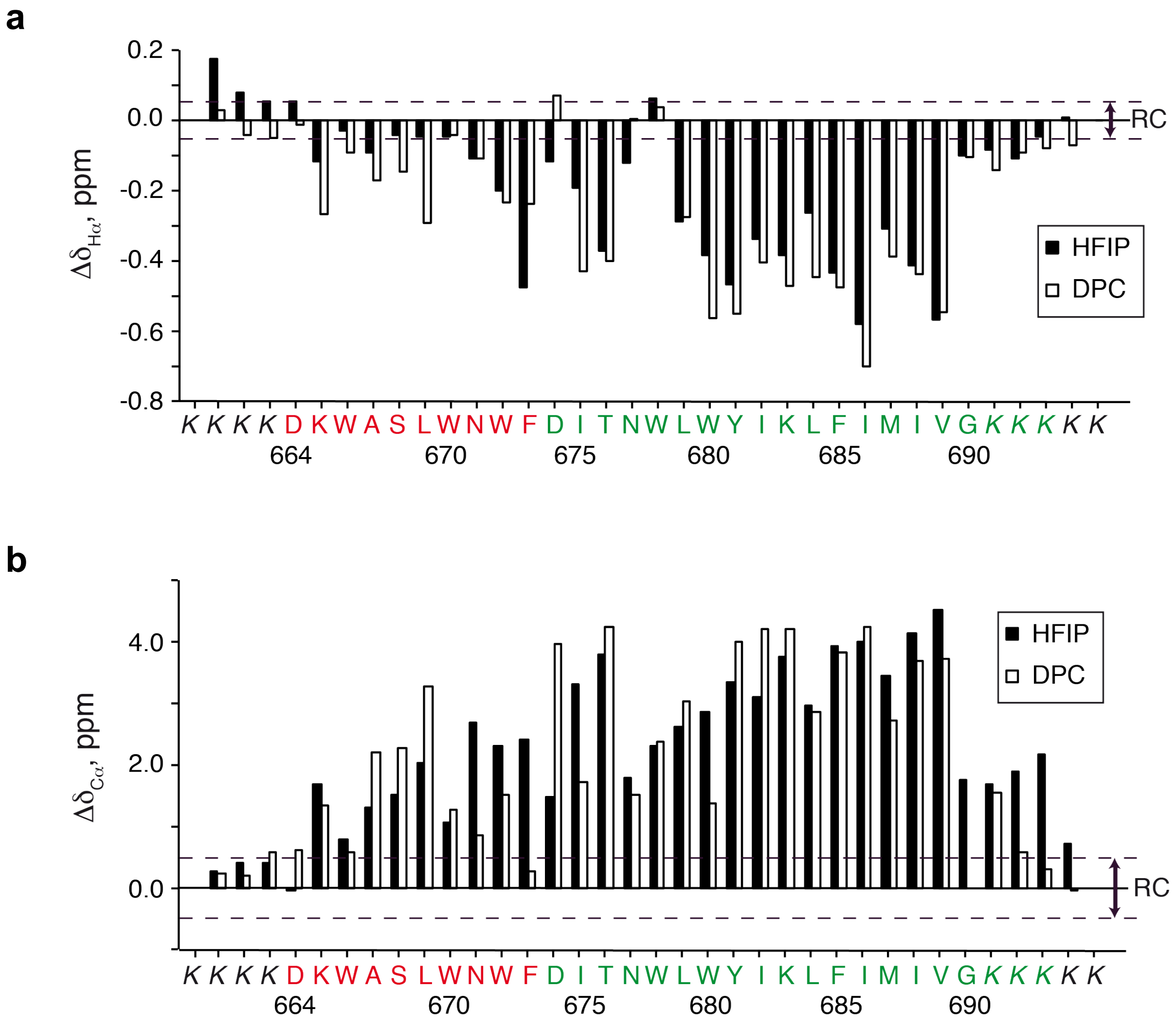

Supplementary Figure 1: NMR parameters for 10E8ep. Bar graphs showing $\Delta\delta_{H\alpha}$ ($\Delta\delta_{H\alpha} = \delta_{H\alpha}^{observed} - \delta_{H\alpha}^{RC}$, ppm) and $\Delta\delta_{C\alpha}$ ($\Delta\delta_{C\alpha} = \delta_{C\alpha}^{observed} - \delta_{C\alpha}^{RC}$, ppm) values (**(a)** and **(b)** panels, respectively) as a function of sequence in 25 % HFIP (black bars) or 20 mM DPC (white bars) at pH 7.0 and 35ºC. Negative ΔδHα values of large magnitude ($|\Delta\delta_{H\alpha}| > 0.05$ ppm) and large positive ΔδCα values ($|\Delta\delta_{C\alpha}| > 0.5$ ppm) are indicative of helical conformations. Residues belonging to helix H1 and H2 are in red and green, respectively, and those of the solubility-tags are in italics. Dashed lines indicate the random coil (RC) ranges. Random coil values for H_α protons and C_α carbons were taken from Wishart et al.[22]. The N- and C-terminal residues are excluded because of charged end effects.

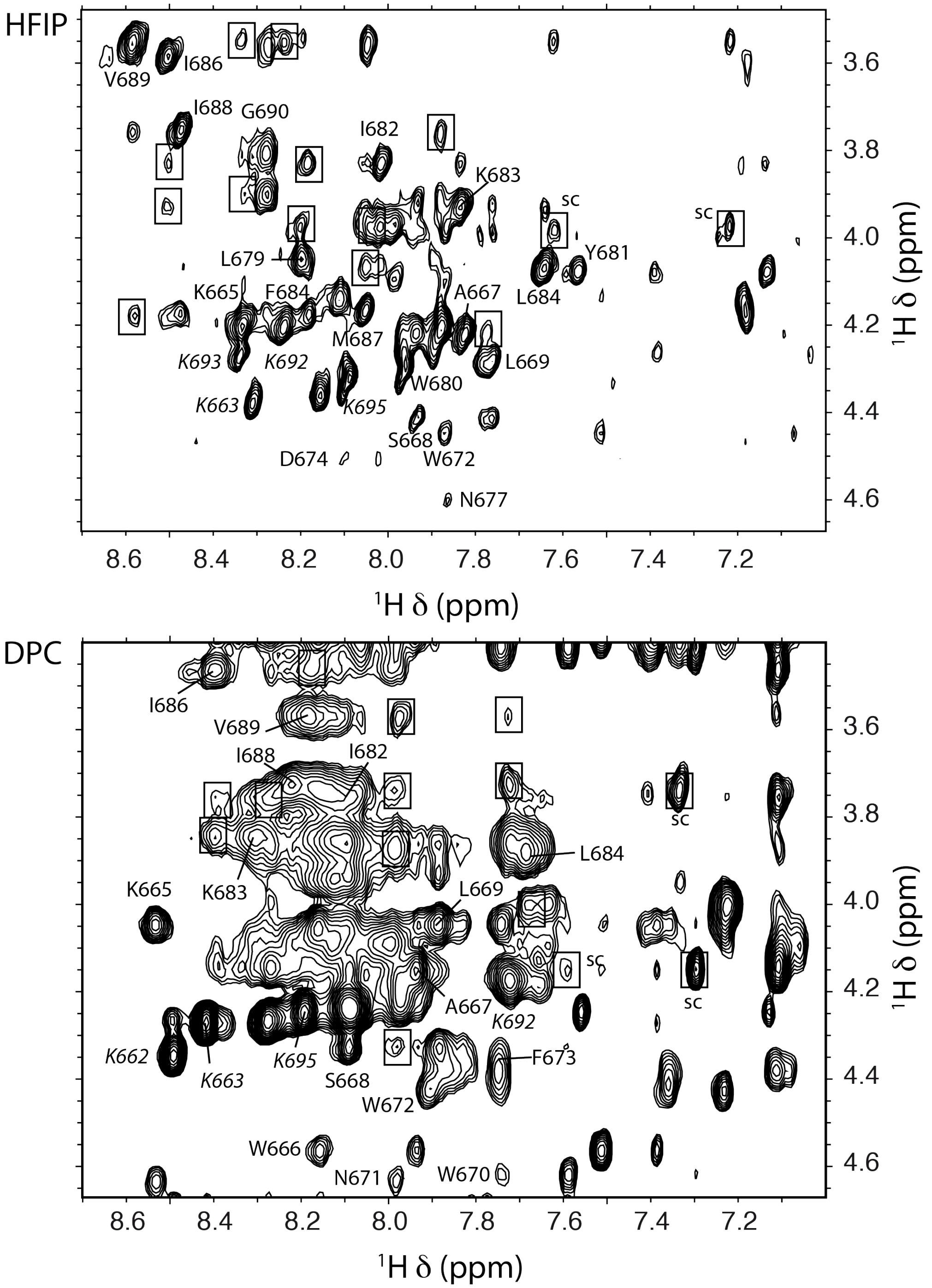

Supplementary Figure 2: Selected regions of the 2D ^{1}H,^{1}H-NOESY spectra recorded for E108p. Spectra were obtained in 25 % HFIP (top) and in 20 mM DPC (bottom) at pH 7.0 and 35ºC. In the less crowded regions, the intra-residual dαN(i,i) NOEs are labeled, and the medium-range non-sequential NOEs are boxed. Residues belonging to the solubility-tags are in italics. 'sc' indicates the cross-peaks corresponding to non-sequential NOEs involving side chain protons. All the other non-sequential cross-peaks are dαN(i,i+2), dαN(i,i+3), or dαN(i,i+4), all of them characteristic of helical structures.

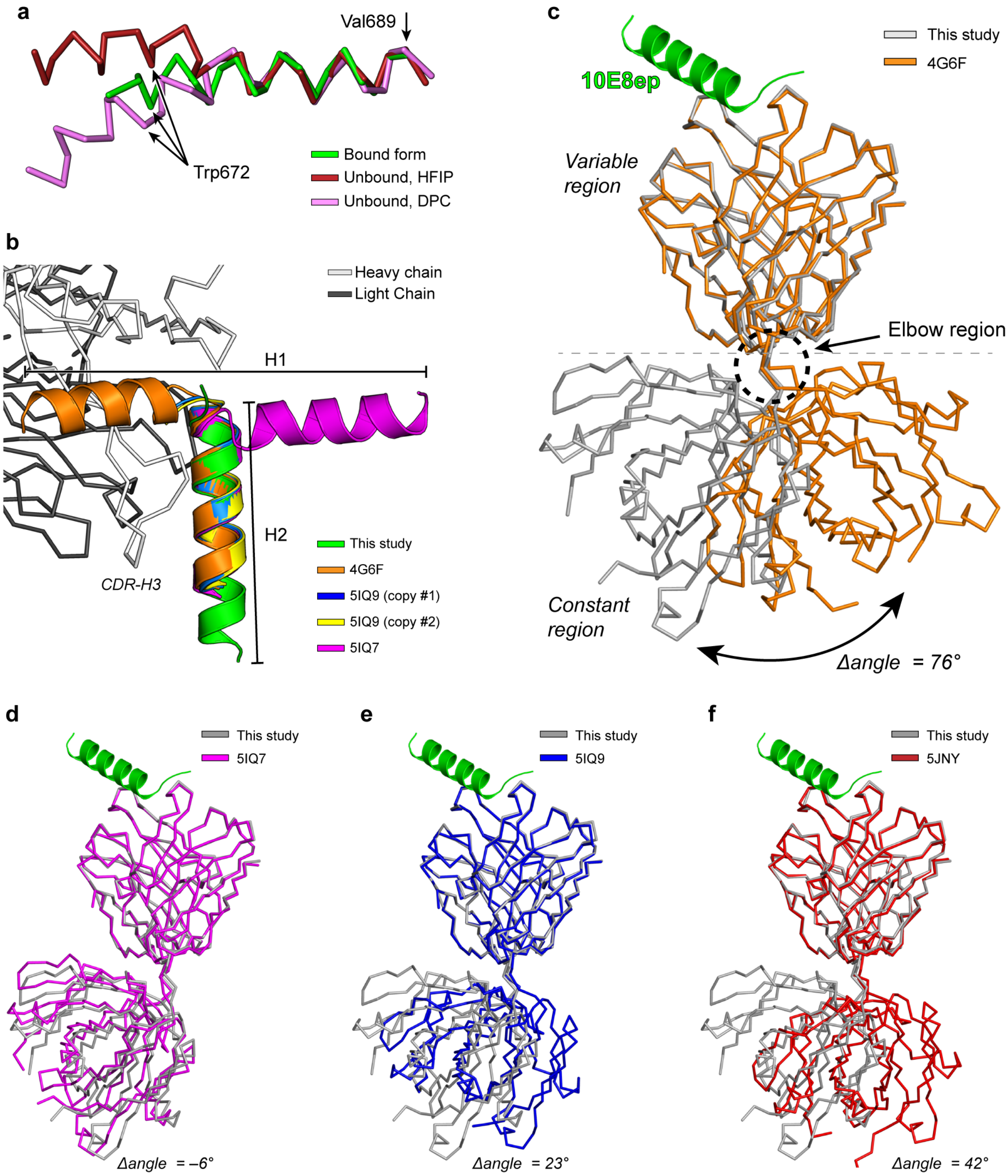

Supplementary Figure 3: Binding properties of 10E8. (a) Superposition of the structure of the peptide in solution (violet and red-brown) and bound to 10E8 (green). Peptides are depicted as ribbons. **(b)** Comparison of the conformation of the peptide bound to 10E8 Fab in multiple crystal structures. The peptide bound to the Fab in this study and in PDB entry codes 4G6F, 5IQ9 (two copies) and 5IQ7 are shown in green, orange, blue, yellow and magenta, respectively. Residues of the variable region (10-90) of the light chain were employed for superposition of the structures, achieving RMSD values in the range of 0.19-0.34 Å. The region H1 appears in

multiple conformations or it is not observed, whereas H2 remains essentially unchanged. The crystal structure of the Fab determined in this study is depicted with light (heavy chain) and gray (light-chain) ribbons. **(c)** Domain rotation (elbow angle). Comparison of two structures of 10E8 Fab with peptide bound. Gray and magenta ribbons correspond to the structure determined in this study and with PDB entry code 4G6F, respectively. The angle of the constant region with respect to the variable region shifts by 76° between the two structures, producing a conformational change (swinging-like) that is clearly visible in the figure. The elbow angles were calculated with the program PHENIX. For clarity purposes only the peptide 10E8ep bound to the Fab is shown. **(d-f)** Equivalent representation comparing the structure of 10E8 bound to 10E8 and other crystal structures of 10E8. The PDB entry code of each structure is given in the panel.

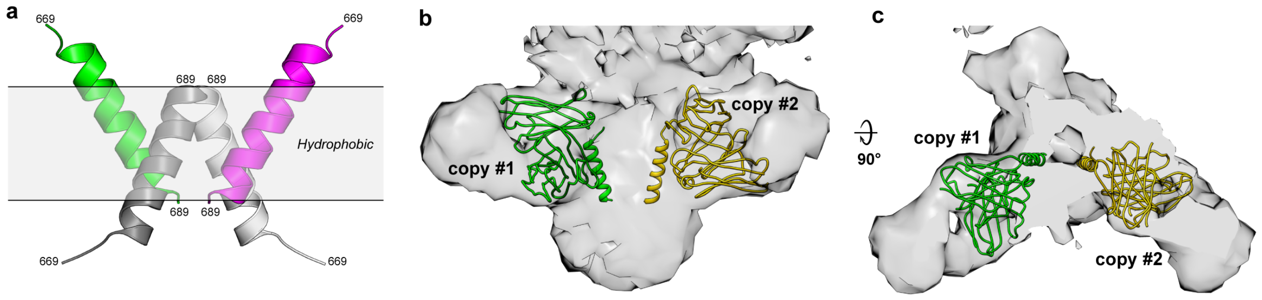

Supplementary Figure 4: Intermolecular peptide-peptide interactions in the crystal. (a) Close-up view of several peptides arranged in a micelle-like aggregate in the crystal structure of 10E8ep bound to Fab. The micelle-like aggregate is stabilized by numerous non-polar interactions between hydrophobic residues of the peptide. The micelle-like aggregate mimics the environment of a biological membrane, and is presumably surrounded by detergent molecules (DPC) from the crystallization buffer, although we note that no molecules of detergent were observed in the electron density. The gray box indicates the approximate region occupied by the nonpolar residues. **(b, c)** Two views showing the X-ray structure of the variable region of 10E8 in complex with 10E8ep fitted into the electron density map of EnvΔCT-PGT151-10E8 (Entry code EMD-3312). Only the Fv residues are fitted, consistent with the procedure in Lee et al [23]. Superposing other structures of 10E8 renders essentially the same conformation as already shown in Figure S3b.

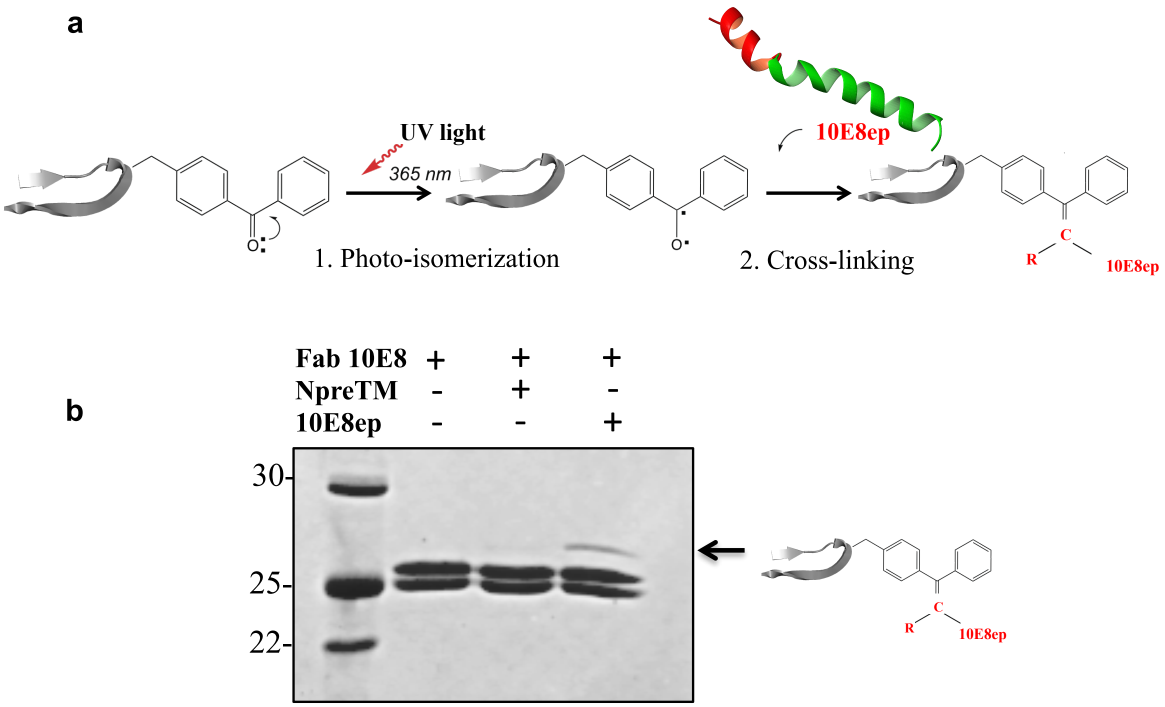

Supplementary Figure 5: Photo-cross-linking assay using 10E8 Fab that incorporates genetically encoded photoactivatable amino acid, p-benzoyl-L-phenylalanine (pBPA) at position 100b$_{HC}$. (a) Chemical structure and schematic mechanism of covalent bond formation between Fab-pBPA and peptide upon irradiation with u.v. light. **(b)** Assay illustrating cross-linking of 10E8 Fab-pBPA with peptide epitope. A band corresponding to the Fab HC-peptide adduct was observed in the irradiated samples containing 10E8ep (indicated by the arrow), but not in control samples without peptide, or in samples that contained the MPERp peptide ending at Lys683 [21]. Protein bands were solved by SDS-PAGE and visualized by staining with Coomassie.

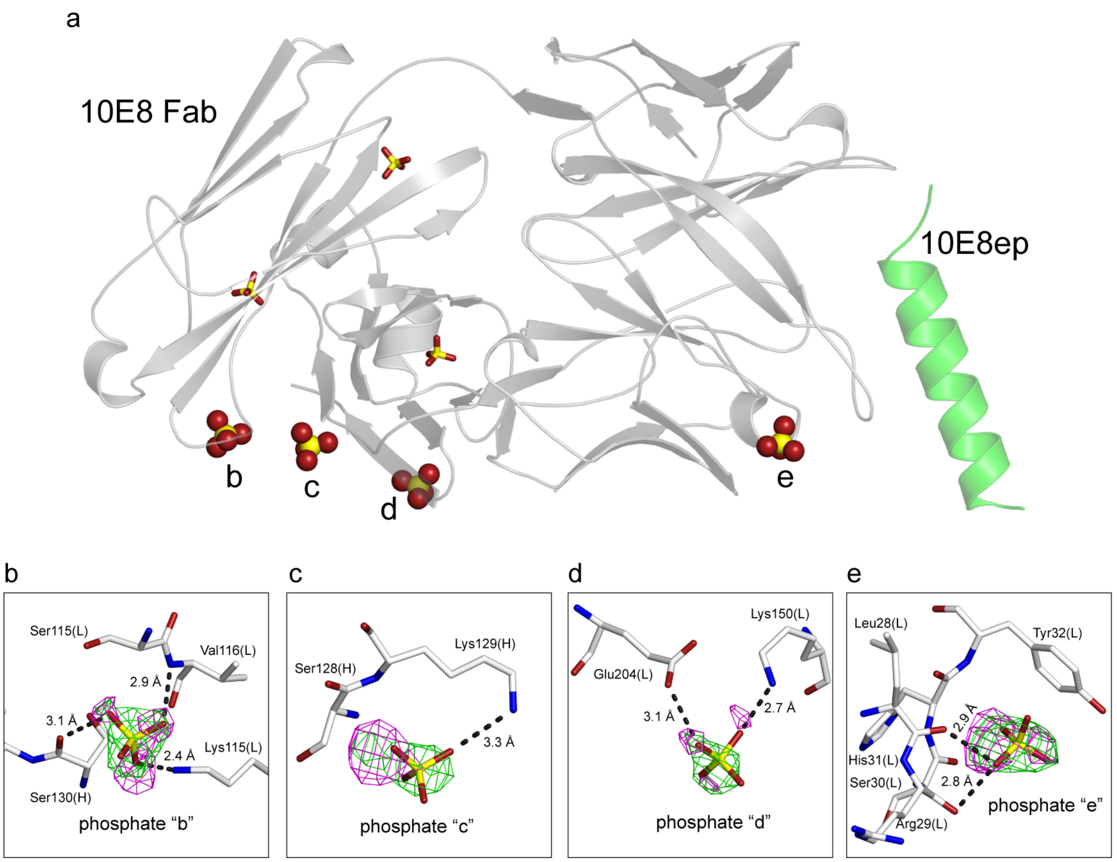

Supplementary Figure 6: Electron density features of phosphate ions located on the surface of 10E8. (a) Depiction of four different phosphate ions (labeled b-e) at the putative membrane-Fab interface. Three other phosphates in regions far from the putative membrane-Fab interface are depicted with sticks. The orientation of Fab is the same as in Figure 4a of the manuscript. These phosphates were modeled based on five conditions: (i) The difference electron density fo-fc and 2fo-fc maps showed strong electron density features before the phosphate moiety was modeled. Typical σ values for the fo-fc map were 4.7-8.2. Typical σ values for the 2fo-fc map were 2.0-3.3. (ii) When modeled, the phosphate ions engaged in non-covalent interactions with the neighboring residues. (iii) A water molecule could not properly account for the electron density (see below). (iv) A water molecule modeled at the position of the phosphorous atom could not engage in H-bonds with neighboring residues. (v) Other molecules present in the solution like TRIS, 2-methyl-2,4-pentanediol, DPC, or glycerol did not seem to fit the electron density. **(b-e)** OMIT difference electron density map at the location of each phosphate is shown as a green mesh. Difference fo-fc electron density map (magenta mesh) was obtained by placing a water molecule instead of a phosphate ion. The water molecule is initially placed at the position of the phosphorous atom and subsequently refined with REFMAC5. Maps are shown at a contouring level of σ = 3.

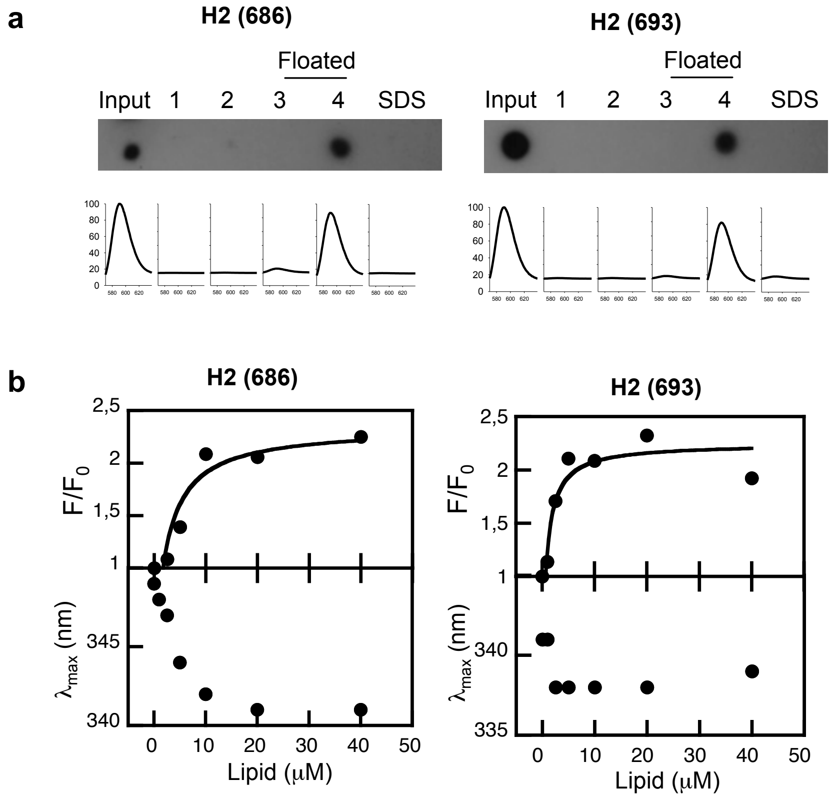

Supplementary Figure 7: Peptide-liposome complex formation. (a) Incorporation of H2(686) or H(693) into liposomes was determined after flotation of the complexes in a sucrose gradient. N-Rh-PE-labeled liposomes and peptides were incubated at the same concentrations as in photo-cross-linking assays (1.5 mM and 10 μM, respectively). 100 μl of the mixture was adjusted to a sucrose concentration of 1.4 M in a final volume of 300 μl, and subsequently overlaid with 400 and 300 μl-layers of 0.8 and 0.5 M sucrose, respectively. The gradient was centrifuged at 436,000 x g for 3 h in a TLA 120.2 rotor (Beckman Coulter, Brea CA, USA). After centrifugation, four 250 μl-fractions were collected. Material adhered to the tubes was collected into a 5th fraction by washing with 250 μl of hot (100º C) 1% (w/v) SDS. The presence of peptides or liposomes was revealed by dot-blot analysis or rhodamine fluorescence (top and bottom panels, respectively). Peptides and liposomes were recovered from the floating fraction number 4, confirming full and irreversible incorporation of the peptides into vesicles. **(b)** Titration experiments following the changes in peptide Trp fluorescence intensity that occur upon membrane insertion. Changes in fractional intensity (top) and maximum emission wavelength (bottom) were measured upon incubation with increasing concentrations of lipid vesicles. Peptide concentration was 0.5 μM. The lines correspond to the best fit of the experimental values to a hyperbolic function. Saturation values indicate that quantitative incorporation of peptide can be attained with lipid concentrations higher than 50 μM.

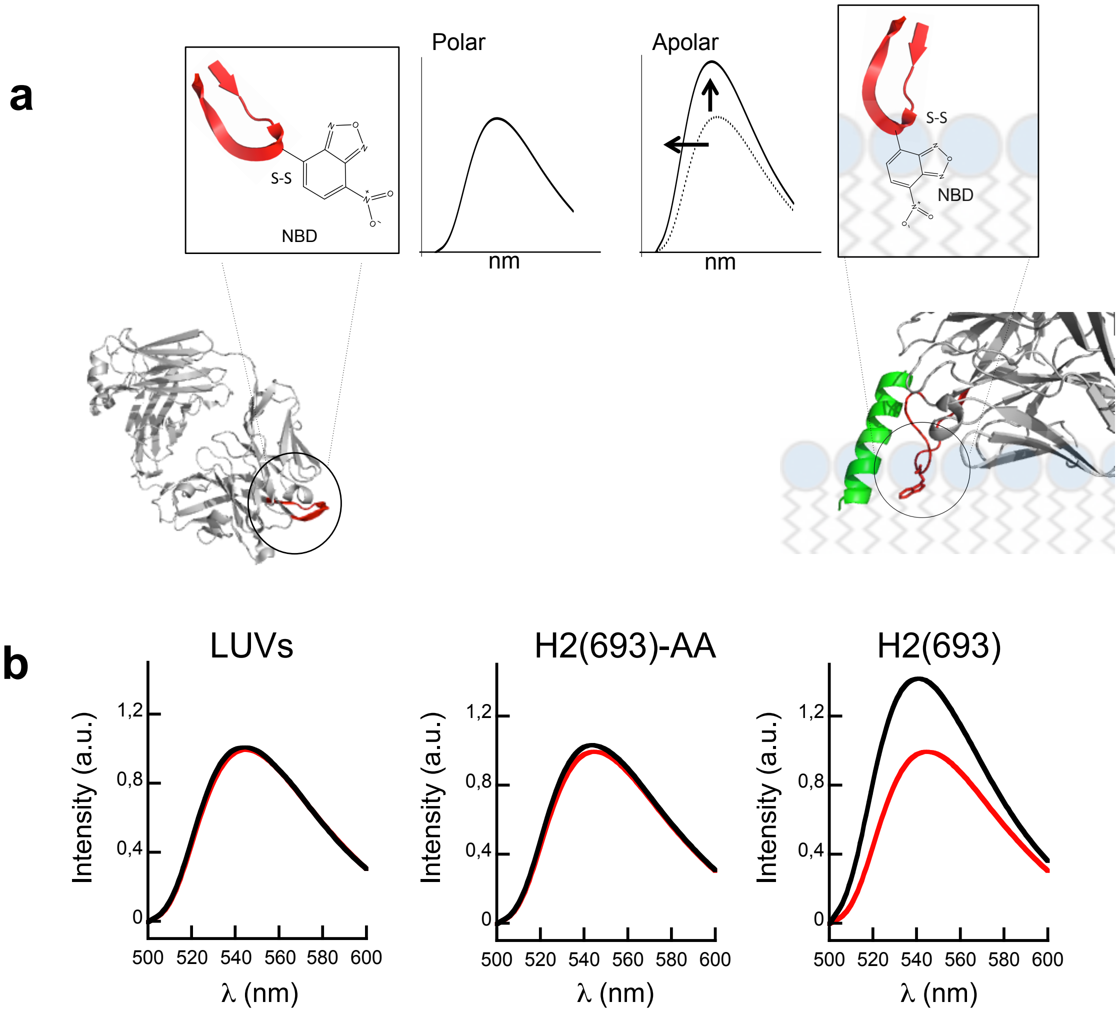

Supplementary Figure 8: Membrane penetration of the fluorophore 7-nitrobenz-2-oxa-1,3-diazol-4-yl (NBD) covalently attached to 10E8 Fab. (a) Strategy of labeling and spectroscopic properties of the NBD-Fab in solution or upon membrane insertion. **(b)** Emission spectra of the labeled 10E8 NBD-Fab (0.5 μM) obtained in presence of bare liposomes (left), liposomes containing H(693)-AA (center) or liposomes containing H(693) (right). Lipid and peptide concentrations were 250 and 6,8 μM respectively. In H(693)-AA sequence key epitope residues Trp672/Phe673 were substituted by Ala. Red traces correspond to spectra obtained in solution with maximum emission normalized to 1.

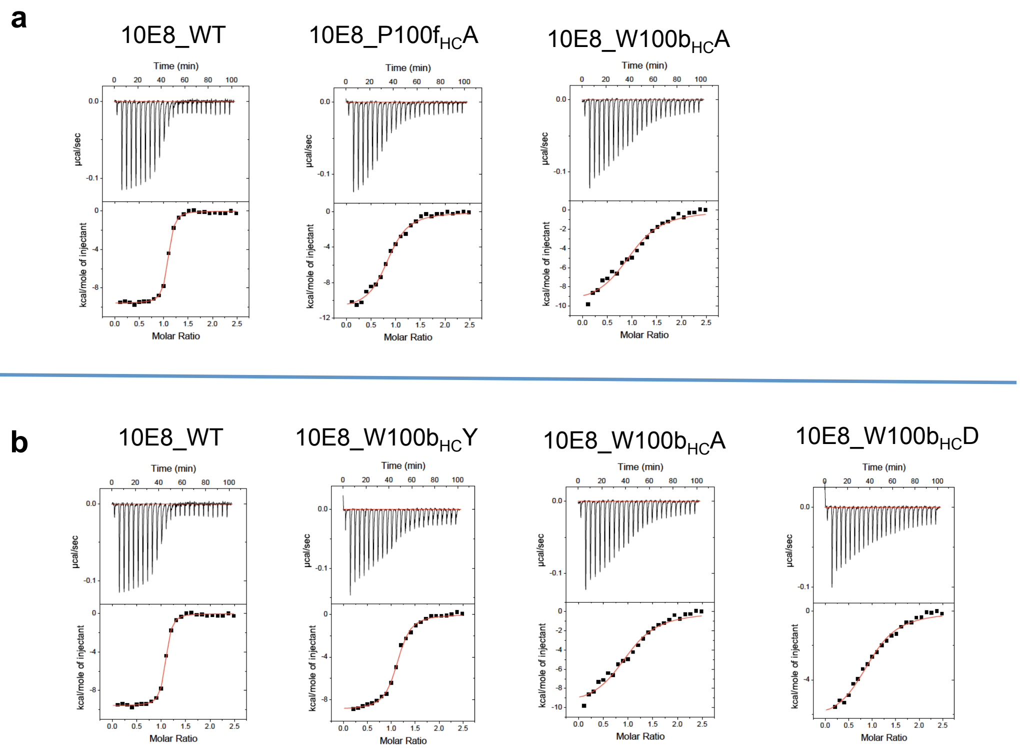

Supplementary Figure 9: 10E8ep peptide binding to CDR H3 10E8 Fab mutants by ITC. (a) Binding of Fab mutants with Ala substitutions P100f$_{HC}$A and W100b$_{HC}$A, reflecting higher affinity of the former (Table 2), and correlating with its higher neutralization potency (Fig. 5c). **(b)** Effect of substitutions at position 100b$_{HC}$ critical for neutralization. The Fab containing the conservative W100b$_{HC}$Y substitution displayed the highest affinity (Table 2), which also correlated with its comparatively higher neutralizing activity (Fig. 5c).

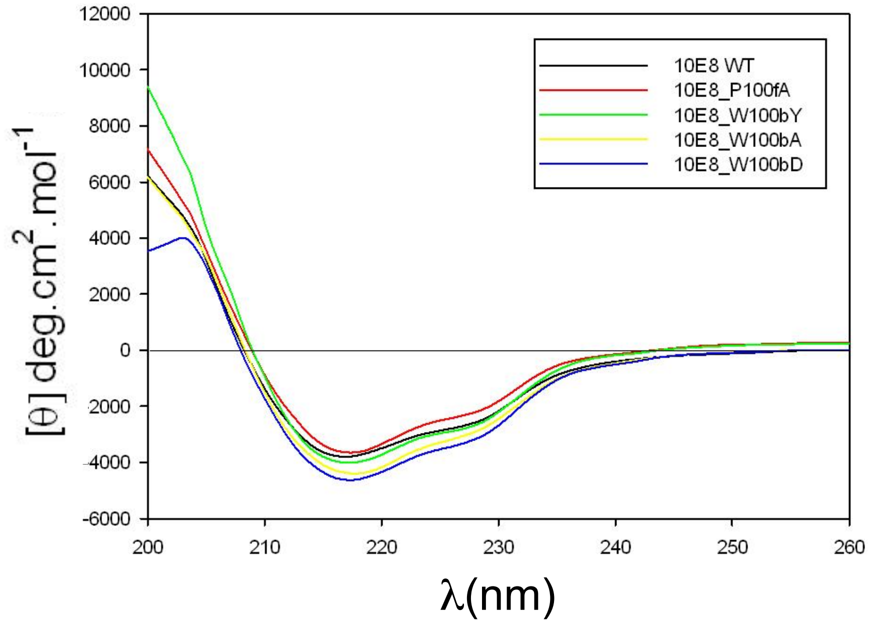

Supplementary Figure 10: Circular Dichroism spectra of 10E8 Fab and its CDR H3 mutants. Measurements were done in PBS at 25 ºC. Fab concentration was 30 μM in all samples.

Supplementary Table 1. Structural statistics for the ensemble of the 20 lowest target function NMR structures of 10E8ep in DPC (20 mM deuterated dodecylphosphocholine, 2 mM HEPES pH 7.0, H_2O/D_2O 9:1 v/v) and HFIP (25 % deuterated 1,1,1,3,3,3-hexafluoro-2-propanol in 2 mM HEPES pH 7.0, H_2O/D_2O 9:1 v/v). PDB codes are indicated in parenthesis.

	**HFIP (2NCT)**	**DPC (2NCS)**
**Number of distance restraints**		
Intraresidue (i – j = 0)	182	198
Sequential (|i – j| = 1)	97	113
Medium range (1 < |i – j| < 5)	82	134
Total number	361	445
Averaged total number per residue	10.0	12.4
**Number of dihedral angle constraints**		
ϕ angles	29	26
ψ angles	29	26
Total number	58	52
**Average maximum violations per structure**		
Distance (Å)	0.02 ± 0.00	0.03 ± 0.02
Dihedral angle (º)	0.05 ± 0.00	0.4 ± 0.2
**Averaged CYANA target function value**	0.003 ± 0.0001	0.11 ± 0.001
**Number of close contacts**	0	0
**Deviations from ideal geometry**		
Bond length (Å)	0.001	0.001
Bond angle (º)	0.2	0.2
**Pairwise rmsd (Å)**		
Backbone atoms (All heavy atoms)		
All residues	2.7 ± 1.0 (3.8 ± 0.9)	2.7 ± 0.8 (3.7 ± 0.7)
Ordered residues (664-691)	0.7 ± 0.2 (1.6 ± 0.3)	0.4 ± 0.1 (0.9 ± 0.1)
Helix H1 (664-673)	0.5 ± 0.2 (2.1 ± 0.5)	0.16 ± 0.08 (0.6 ± 0.2)
Helix H2 (675-691)	0.25 ± 0.08 (0.7 ± 0.2)	0.17 ± 0.06 (0.7 ± 0.1)
**Ramachandran plot (%)**		
All residues (Ordered residues: 664-691)		
Residues in most favoured regions	91.8 (100.0)	88.8 (100.0)
Residues in additional allowed regions	6.7 (0.0)	8.6 (0.0)
Residues in generously allowed regions	1.7 (0.0)	2.4 (0.0)
Residues in disallowed regions	0.5 (0.0)	0.2 (0.0)

Supplementary Table 2:Data collection and refinement statistics (molecular replacement)[a,b]

	10E8 + 10E8ep
**Data collection**	
Space group	I 2 2 2
Cell dimensions	
a, *b*, *c* (Å)	66.6, 81.2, 254.2
α, β, γ (°)	90.0, 90.0, 90.0
Resolution (Å)	52.4 - 2.40 (2.49 - 2.40)
R_{merge}	4.6 (20.2)
$I / \sigma I$	34.0 (8.0)
$CC_{1/2}$	0.999 (0.971)
Completeness (%)	90.0 (56.2)
Redundancy	9.7 (7.0)
**Refinement**	
Resolution (Å)	52.4 – 2.40
No. reflections	24,462
R_{work} / R_{free}	19.2 / 23.4
No. atoms	
Protein	3,360
Peptide	201
Ligand/ion	35
Water	132
B-factors	
Protein	40.0
Peptide	59.5
Ligand/ion	30.0
Water	48.2
R.m.s. deviations	
Bond lengths (Å)	0.011
Bond angles (°)	1.3

[a]Data obtained from a single crystal.
[b]Values in parentheses are for highest-resolution shell.

Supplementary Table 3: Hydrogen-bond connectivity between 10E8 and two distinct peptides.

Peptide	10E8	10E8ep	MPER[a]
		Distance (Å)	
Trp670	H-Glu53	-	2.8
Trp672	H-Glu53	3.0	3.1[b]
Trp672	H-Pro100G	2.8	2.9
Lys683[c]	H-Phe100A	3.0	3.0
Lys683[c]	H-Gly100D	2.7	2.9

[a] Peptide in complex with Fab in PDB structure 4G6F
[b] Only observed in one copy
[c] This residue is an Arg in the structure of 10E8 with MPER

Supplementary Table 4: Interaction surface of the light chain of 10E8 Fab with two distinct peptides.

Residue	10E8ep	MPER[a]
	BSA (Å^2)	
L-Arg91	15.7	16.3
L-Arg95B	69.3	49.9
Subtotal	*85.0*	*66.3*
Pep-Asn671	1.5	-
Pep-Trp672	10.4	10.9
Pep-Phe673	63.7	54.6
Pep-677	1.0	-
Subtotal	*76.6*	*65.4*
**Total**	**161.6**	**131.7**

[a] Peptide in complex with Fab in PDB structure 4G6F

Supplementary Table 5: Interaction surface of the heavy chain of 10E8 Fab with two distinct peptides.

	**10E8ep**	**MPER[a]**
**Residue**	**BSA (Å^2)**	
H-Asp28	-	14.4
H-Asn31	16.6	33.2
H-Trp33	27.3	29.2
H-Arg50	7.9	5.9
H-Thr52	4.9	4.6
H-Pro52B	29.9	23.8
H-Gly52C	52.8	58.5
H-Glu53	59.0	59.7
H-Ser56	9.5	7.1
H-Asp58	3.6	6.9
H-Lys97	53.2	46.1
H-Tyr98	3.1	4.1
H-Tyr99	80.1	81.4
H-Phe100A	98.4	80.1
H-Trp100B	63.2	32.2
H-Ser100C	1.4	5.1
H-Gly100D	16.7	16.2
H-Tyr100E	32.0	25.3
H-Pro100F	67.7	65.5
H-Pro100G	63.6	62.1
H-Gly100H	-	0.1
H-Glu100I	0.6	1.4
H-Glu100J	4.5	5.3
Subtotal	*695.8*	*668.3*
Pep-Leu-661[b]	-	1.8
Pep-Asp664	-	0.2
Pep-Lys665	-	22.0
Pep-Ser668	-	53.9
Pep-Leu669	67.4	28.2
Pep-Trp670	41.4	11.7
Pep-Asn671	27.8	34.3
Pep-Trp672	129.3	134.2
Pep-Phe673	84.4	94.1
Pep-Thr676	49.2	48.4
Pep-Asn677	17.0	19.6
Pep-Leu679	51.0	46.2
Pep-Trp680	66.1	64.3
Pep-Ile682	6.0	15.0
Pep-Lys683[c]	84.0	106.8
Pep-Ile686	29.8	-
Pep-Met687	19.6	-
Subtotal	*672.9*	*680.5*
**Total**	**1368.7**	**1348.8**

[a] Peptide in complex with Fab in PDB structure 4G6F
[b]This residue is not present in 10E8ep
[c]This residue is an Arg in the structure of 10E8 with MPER

Supplementary References:

1. Markley JL, Bax A, Arata Y, Hilbers CW, Kaptein R, et al. (1998) Recommendations for the presentation of NMR structures of proteins and nucleic acids. J Mol Biol 280: 933-952.
2. Mirassou Y, Santiveri CM, Perez de Vega MJ, Gonzalez-Muniz R, Jimenez MA (2009) Disulfide bonds versus TrpTrp pairs in irregular beta-hairpins: NMR structure of vammin loop 3-derived peptides as a case study. ChemBioChem 10: 902-910.
3. Markley JL, Bax A, Arata Y, Hilbers CW, Kaptein R, et al. (1998) Pure Appl Chem 70: 117-142.
4. Guntert P, Mumenthaler C, Wuthrich K (1997) Torsion angle dynamics for NMR structure calculation with the new program DYANA. J Mol Biol 273: 283-298.
5. Guntert P (2004) Automated NMR structure calculation with CYANA. Methods Mol Biol 278: 353-378.
6. Laskowski RA, Rullmannn JA, MacArthur MW, Kaptein R, Thornton JM (1996) AQUA and PROCHECK-NMR: programs for checking the quality of protein structures solved by NMR. J Biomol NMR 8: 477-486.
7. Koradi R, Billeter M, Wuthrich K (1996) MOLMOL: a program for display and analysis of macromolecular structures. J Mol Graph 14: 51-55, 29-32.
8. Evans P (2006) Scaling and assessment of data quality. Acta Crystallogr Sect D 62: 72-82.
9. Evans PR, Murshudov GN (2013) How good are my data and what is the resolution? Acta Crystallogr Sect D 69: 1204-1214.
10. Huang J, Ofek G, Laub L, Louder MK, Doria-Rose NA, et al. (2012) Broad and potent neutralization of HIV-1 by a gp41-specific human antibody. Nature 491: 406-412.
11. McCoy AJ, Grosse-Kunstleve RW, Adams PD, Winn MD, Storoni LC, et al. (2007) Phaser crystallographic software. J Appl Crystallogr 40: 658-674.
12. Emsley P, Lohkamp B, Scott WG, Cowtan K (2010) Features and development of Coot. Acta Crystallogr D 66: 486-501.
13. Murshudov GN, Vagin AA, Dodson EJ (1997) Refinement of macromolecular structures by the maximum-likelihood method. Acta Crystallogr D 53: 240-255.
14. Laskowski RA, MacArthur MW, Moss DS, Thornton JM (1993) PROCHECK - a program to check the stereochemical quality of protein structures. J App Cryst 26: 283-291.
15. Krissinel E, Henrick K (2007) Inference of macromolecular assemblies from crystalline state. J Mol Biol 372: 774-797.
16. Lawrence MC, Colman PM (1993) Shape complementarity at protein-protein interfaces. J Mol Biol 234: 946-950.
17. Stanfield RL, Zemla A, Wilson IA, Rupp B (2006) Antibody elbow angles are influenced by their light chain class. J Mol Biol 357: 1566-1574.
18. Irimia A, Sarkar A, Stanfield RL, Wilson IA (2016) Crystallographic Identification of Lipid as an Integral Component of the Epitope of HIV Broadly Neutralizing Antibody 4E10. Immunity 44: 21-31.
19. Pettersen EF, Goddard TD, Huang CC, Couch GS, Greenblatt DM, et al. (2004) UCSF Chimera--a visualization system for exploratory research and analysis. J Comput Chem 25: 1605-1612.

20. Montero M, Gulzar N, Klaric KA, Donald JE, Lepik C, et al. (2012) Neutralizing epitopes in the membrane-proximal external region of HIV-1 gp41 are influenced by the transmembrane domain and the plasma membrane. J Virol 86: 2930-2941.
21. Serrano S, Araujo A, Apellaniz B, Bryson S, Carravilla P, et al. (2014) Structure and immunogenicity of a peptide vaccine, including the complete HIV-1 gp41 2F5 epitope: implications for antibody recognition mechanism and immunogen design. J Biol Chem 289: 6565-6580.
22. Wishart DS, Bigam CG, Holm A, Hodges RS, Sykes BD (1995) 1H, 13C and 15N random coil NMR chemical shifts of the common amino acids. I. Investigations of nearest-neighbor effects. J Biomol NMR 5: 67-81.
23. Lee JH, Ozorowski G, Ward AB (2016) Cryo-EM structure of a native, fully glycosylated, cleaved HIV-1 envelope trimer. Science 351: 1043-1048.
